# Circ-0044539 promotes lymph node metastasis of hepatocellular carcinoma through exosomal-miR-29a-3p

**DOI:** 10.1038/s41419-024-07004-x

**Published:** 2024-08-27

**Authors:** Yi Yang, Xue-Qin Chen, Ya-Xun Jia, Jie Ma, Di Xu, Zuo-Lin Xiang

**Affiliations:** 1 Department of Radiation Oncology, Shanghai East Hospital, School of Medicine, Tongji University, Shanghai, 200120 China; 2 Department of Radiation Oncology, Shanghai East Hospital Ji’an hospital, Ji’an City, Jiangxi Province 343000 China

**Keywords:** Tumour biomarkers, Tumour immunology

## Abstract

Lymph node metastasis **(**LNM) is a common invasive feature of hepatocellular carcinoma (HCC) associated with poor clinical outcomes. Through microarray profiling and bioinformatic analyses, we identified the circ-0044539-miR-29a-3p-VEGFA axis as a potential key factor in the progression of HCC LNM. In HCC cells and nude mice, circ-0044539 downregulation or miR-29a-3p upregulation was associated with small tumor size, PI3K-AKT-mTOR pathway inactivation, and downregulation of the key LNM factors (HIF-1α and CXCR4). Furthermore, circ-0044539 was also responsible for exosomal miR-29a-3p secretion. Exosomal miR-29a-3p was then observed to migrate to the LNs and downregulate High-mobility group box transcription factor 1 (Hbp1) in Polymorphonuclear Myeloid-derived suppressor cells (PMN-MDSCs), inducing the formation of a microenvironment suitable for tumor colonization. Overall, circ-0044539 promotes HCC cell LNM abilities and induces an immune-suppressive environment in LNs through exosomes, highlighting its potential as a target for HCC LNM and HCC immunotherapy.

## Introduction

Hepatocellular carcinoma (HCC) is the most common primary liver tumor with high global incidence, which ranks second in terms of cancer-related mortality, preceded only by pancreatic cancer [1]. LNM is one of the most common types of extrahepatic metastasis in HCC and greatly impacts the survival and quality of life of HCC patients. Despite remarkable improvements in HCC diagnosis and treatment, detecting LNM is difficult due to mild symptoms. Furthermore, the specific biomarker and underlying mechanism of HCC LNM are still unknown. Therefore, there is an urgent need to comprehensively clarify the molecular mechanisms involved in LNM to improve intervention and diagnosis strategies for HCC patients.

Circular RNAs (circRNAs), due to their unique circular structure, exhibit high conservation and stability, which enables them to serve as potential molecular biomarkers in serum. CircRNAs were found to be associated with diverse mechanisms underlying HCC pathogenesis, including the formation of immunosuppressive environments, angiogenesis, matrix remodeling, hypoxia, release of extracellular vesicles, and resistance to chemoradiotherapy [2, 3]. The enormous potential of circRNAs as noninvasive biomarkers and their clinical value in targeted therapy has garnered widespread attention. However, the specific role of circRNAs in HCC LNM is unknown. This study aims to validate the biological role of circ-0044539 in HCC and explore the molecular mechanism of its involvement in the process of HCC LNM, to provide theoretical support for the development of novel clinical diagnostic markers and therapeutic targets for HCC LNM patients.

Myeloid-derived suppressor cells (MDSCs) are a heterogeneous population of myeloid cells that are generated in the bone marrow. In tumor-bearing hosts, MDSCs migrate to peripheral lymphoid organs and the tumor to contribute to the formation of the local immunosuppressive microenvironment [4]. Remarkable disparities in the phenotypes of MDSCs isolated from various cancer tissues and organs have been identified, indicating that the differentiation and accumulation of MDSCs might be cancer-cell dependent. This suggests that MDSCs in blood and immune organs, such as LNs, could be regulated by cancer cells. Although the suppressive role of MDSCs in LN and in HCC tissues has been confirmed, the regulatory network underlying MDSCs conforming to the pre-metastatic LN niche before HCC arrival remains unexplored.

Reportedly, cancer cells can remodel the target organ microenvironment and induce the formation of pre-metastatic niches via exosomes [5]. Exosomes are small extracellular vesicles that play vital roles in a diverse set of physiological and pathological processes, such as the regulation of immune responses and stimulation of tumor progression. A suppressive immune microenvironment is important for LNM [6]. MiRNAs present in exosomes have been suggested as potential biomarkers for cancer. Hbp1 is a transcriptional repressor that inhibits the G1 phase of the cell cycle [7]. Both miR-29a-3p overexpression and Hbp1 silencing increased the production of Arg1 and NO in MDSCs [8]. We hypothesized that exosomal miR-29a-3p released by HCC cells serves as a crucial driver of LNM and revealed the molecular mechanisms for shaping the pre-metastatic LN niche. We found that HCC cells with high expression levels of circ-0044539 released exosomes containing miR-29a-3p, which were then taken up by recipient PMN-MDSCs, and these miR-29a-3p targeted Hbp-1 mRNA, resulting in improved functions of PMN-MDSCs and the formation of an immunosuppressive LN microenvironment that promotes LNM.

Overall, this study demonstrates that HCC with high LNM potential is characterized by circ-0044539 which promotes LNM via modulation of HCC metastatic ability and the LN microenvironment. Additionally, this research partly elucidated the immunomodulatory mechanisms of primary HCC lesions. A deeper understanding of LN environmental changes in HCC patients could be instrumental in guiding the rationalized design of improved immunotherapies.

## Methods

### Patients and samples

All HCC tissue samples were procured from patients diagnosed with HCC during surgical procedures conducted between 2018 and December 2022 at the Shanghai East Hospital, Tongji University, China. The selection criteria for HCC cases included clear imaging and pathological diagnosis, along with complete follow-up data. The exclusion criteria included any prior treatment (local or systemic). The tumor stage was determined in accordance with the current American Joint Committee on Cancer/Union for International Cancer Control staging system. The specimens were obtained from patients with their informed consent, in accordance with protocols approved by the institutional review board, and subsequently preserved as required.

### CircRNA array analysis and miRNA analysis

The circRNAs from the serum of HCC patients with or without LNM were collected for microarray analysis. Total RNA was extracted and purified using a Qiagen serum kit according to standard protocols. Then, RNAs were amplified and labeled with the Low Input Quick Amp WT Labeling Kit (Agilent Technologies, US). These labeled RNAs were hybridized using a Gene Expression Hybridization Kit (Agilent Technologies, US), and scanned by an Agilent Microarray Scanner (Agilent Technologies, US). All the reads were first mapped to the human reference genome (GRCh37/hg19). We defined the statistical criteria for selecting differentially expressed circRNAs using *p*-values < 0.05. We analyzed GO terms and KEGG pathways to investigate the possible biological functions of the host genes of these circRNA through the clusterProfiler package [4.4.4](4.2.1).

To identify miRNAs potentially regulated by HCC LNM-associated circRNAs, Circinteractom and miRanda were used to predict targeted miRNAs of COL1A1-related circRNA based on the prediction of miRNA response elements (MREs). Then. we analyzed the target genes of miR-29a-3p via miRWalk, miRanda, starBase, Targetscan. Overlapping genes in all four databases were subjected to STRING protein interaction analysis.

### Exosome isolation and characterization

Exosomes from HCC cells were isolated from a culture medium with exosome-free FBS by using an exosome isolation kit for cell culture media (Yesan, China, 41201ES50). The supernatant collected from 3-day cell cultures was centrifuged at 3000 × *g* at 4 °C for 10 min to remove cell debris. Then, the samples were centrifuged at 10,000 × *g* at 4 °C for 1 h to precipitate exosome sediment according to the manufacture protocol.

Plasma exosomes were purified by using an exosome isolation kit for serum/plasma (Yesan, China, 41202ES30). Briefly, plasma was collected and centrifuged at 3000 × *g* for 10 min. The supernatant was centrifuged at 10,000 × *g* for 20 min. Corresponding amounts of reagents were added to the supernatant, and mixtures were incubated at 4 °C for up to 2 h and then centrifuged at 10,000 × *g* at 4 °C for 1 h to precipitate exosome pellets.

Western blotting was used to confirm exosome protein markers (TSG101). Transmission electron microscopy (TEM) was used to analyze the diameter and shape of exosomes with technical assistance provided by Servicebio Company. The concentration of the exosomes was characterized by NTA with technical assistance provided by Beyotime (China). The quantity of exosomes was determined by BCA assay [9].

### Separation of PMN-MDSCs and T cells

PMN-MDSCs (CD11b+HLADR-CD33 + CD14-CD15 + ) were purified from peripheral blood from HCC patients by Fluorescence-activated Cell Sorting (FACS). T cells (CD3 + CD8 + ) were obtained from peripheral blood mononuclear cells (PBMCs) of healthy donors by FACS separation according to the manufacturer’s instructions. Peripheral blood from HCC patients or PMBC from health donors was counted and then stained with fluorochrome-conjugated anti-human antibodies for 30 min (antibody details are shown in Supplementary Table S2). Using a FACS Aria II flow cytometer (BD Biosciences), the stained cells were sorted into PMN-MDSCs (HLADR-CD11b + CD14-CD33 + CD15 + ), or CD8 + T cells (CD3 + CD8 + ). The PMN-MDSCs and CD8 + T-cell gating strategies are provided in Fig. S5.

PMN-MDSCs were cultured with G-CSF (5 μg/mL) in 10% FBS 1640 within 1 week. CD8 + T cells were cultured with human T-Activator CD3/CD28 (2 μL per 1 × 10^5 ^T cells, a bead-to-cell ratio of 1:1 for short-term of approximately 3 days) in 10% FBS 1640. Beads were removed by a DynaMag™-5 magnetic frame (Invitrogen, USA).

### Co-culture system

Exosomes were extracted from the medium of HCC cell lines, and stained with the green fluorescent dye PKH67 (Umibio, China) for 1 h at 37 °C. Then, these PKH67-labeled exosomes were extracted again to remove the dye. Purified exosomes were co-cultured with PMN-MDSCs in a 6-well cell culture cluster for 24 h, and internalization was determined by fluorescence microscopy.

PMN-MDSCs and T cells were co-cultured indirectly in a 24-well transwell plate at a ratio of 1:4. While treated or untreated PMN-MDSCs were added into the outer chamber, the CD8 + T cells were cultured in the inner chamber with Human T-Activator CD3/CD28. 24 h later, CD8 + T cells in the inner chamber were removed to examine the vitality by LDH release assay and the proliferation ability by CFSE assay. All experiments were performed in triplicate.

### Carboxyfluorescein succinimidyl ester (CFSE) staining assay

T cells isolated from 6 mL of PBMCs from healthy donors by flow sorting were CFSE-labeled (3 μM, Sigma) and seeded in 24-well plates with PMN-MDSCs isolated previously (per well with 1 × 10^6^ T cells and 2.5 × 10^5^ PMN-MDSCs, T cell:MDSC = 4:1). T-cell proliferation was induced by anti-CD3/CD28 stimulation beads (Invitrogen, CA) [10]. After three days of co-culture, T-cell proliferation was analyzed by flow cytometry. Controls included a positive T-cell proliferation control (T cells alone) and induction negative control (T cells culture with PMN-MDSCs only). The number of T cells was counted under a microscope. Samples were also run on a flow cytometer (BD Biosciences, CA), and Beckman software was used to perform data acquisition and analysis. All experiments were performed in triplicate.

### Statistical analysis

Data are presented as the mean ± standard deviation (SD). Pearson’s correlation coefficient analysis was used to analyze the correlations among the indicated factors. One-way ANOVA with Tukey’s method was used for multiple comparisons. Differences between groups were analyzed using Student’s *t*-test or the chi-square test. The association between the circ-0044539 expression level and clinicopathological characteristics was determined using a Chi-square test. TCGA data were from TCGA Research Network: https://www.cancer.gov/tcga. The GEO dataset, available publicly on the NCBI server, was also utilized [11]. A *p* < 0.05 was considered statistically significant.

Additional methods information is provided in Supplementary Materials. All primers and antibodies used in this article are listed in Tables S1–2.

## Results

### Investigation of the key driver circRNA responsible for HCC LNM initiation

**Fig. 1:**
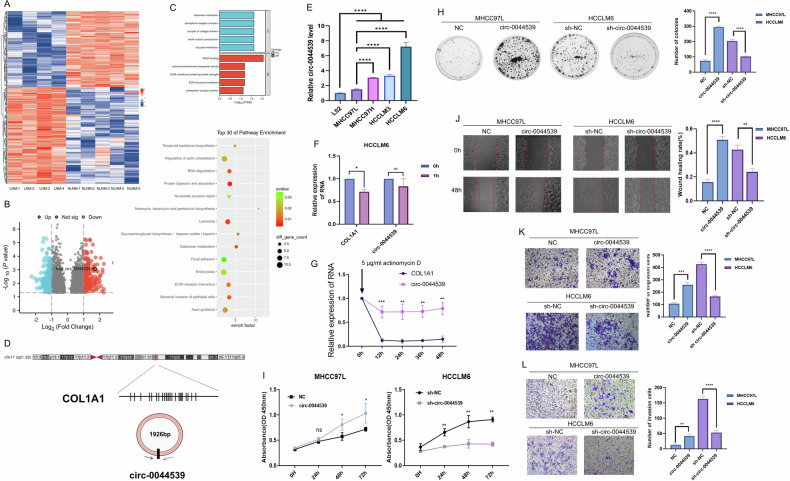
Screening of LNM-related circRNAs in HCC. **A** The cluster heatmap showed the serum levels of 2214 abnormal circRNAs (*p* < 0.05) in HCC LNM (*n* = 4) and HCC NLNM (*n* = 5) patients. **B** Volcano Plot showed the 2214 significantly expressed circRNAs related to HCC LNM. **C** GO and KEGG analyses showed the function enrichment of host genes of 2214 abnormal circRNAs. **D** Presentation of the loop-forming mode of circ-0044539. **E** qRT-PCR showed the expression of circ-0044539 in serial HCC cell lines and the immortalized human normal hepatocyte L02 cell line, normalized to β-actin. **F** The RNA levels of circ-0044539 in HCCLM6 cells after RNase R digestion. (vs. COL1A1 mRNA). **G** The stability of circ-0044539 in HCCLM6 cells after actinomycin D treatment. (vs. COL1A1 mRNA). **H** A monoclonal plate experiment showed the influence of circ-0044539 on the clonogenic ability of MHCC97L and HCCLM6 cells. **I** The CCK-8 assay showed the influence of circ-0044539 on the proliferation ability of MHCC97L and HCCLM6 cells. **J** Wound healing assay showed the influence of circ-0044539 on the migration capacity of MHCC97L and HCCLM6 cells. **K**, **L** Transwell assays without (K) or with (L) matrigel were performed to investigate the influence of circ-0044539 on the migration and invasion capacity of MHCC97L and HCCLM6 cells. Data presented as means ± SD of three independent experiments. *p < 0.05, **p < 0.01, ***p < 0.001, ****p < 0.0001 (Student’s *t*-test).

To define the key circRNA in the progression of HCC LNM, we first collected the serum from HCC patients (LNM: *n* = 4, NLNM: *n* = 5). We used a circRNA array to identify 2214 dysregulated HCC LNM-related circRNAs (*p* < 0.05), among which 380 circRNAs had a fold-change greater than 2 or less than 0.5 (up: 213, down: 167). The expression patterns of these 2214 dysregulated circRNAs are shown in a heatmap and volcano plot (Fig. 1A, B).

CircRNAs typically exhibit functions that align with those of their respective host genes [12]. These host genes of 2214 significantly differentially expressed circRNAs were associated with the GO terms “platelet-derived growth factor (PDGF) binding”, “complex of collagen trimers” and “extracellular matrix (ECM) structural constituent”, and the KEGG terms “ECM-receptor interaction” and “focal adhesion” (Fig. 1C). As reported, these pathways are highly correlated with LNM. For example, ECM degradation leads to loss of integrity in the lymphatic vasculature and subsequent LNM [13]. In addition, the enrichment of “vascular membrane” and “active transmembrane transporter activity” indicated that LNM-related circRNAs might influence HCC LNM by mediating vesicle secretion.

Notably, collagen-related genes were involved in these LNM-related gene sets. Among the 8 collagen-related circRNAs that fulfilled these criteria (*p* < 0.05, ｜log2FC｜≥1), COL1A1 had the highest number of circRNAs (Table 1). Elevated expression of COL1A1 was positively correlated with tumor metastasis and LNM [14, 15]. In TCGA cohorts, COL1A1 mRNA expression was significantly higher in tumor tissues than in adjacent normal tissues across various types of cancer (Fig. S1A). In breast invasive carcinoma (BRCA), prostate adenocarcinoma (PRAD), colon adenocarcinoma (COAD), and bladder cancer (BLCA), COL1A1 expression was higher in the advanced N stage than in the N0 stage (Fig. S1B–E).

**Table 1:** Collagen gene-related circRNA with significant differential expression (fold-change ≥2.0 or ≤0.5, and *p* < 0.05) between HCC LNM and HCC NLNM groups.

circRNA ID	*p*-values	Fold-change	hostgene
hsa_circ_0044534	0.015273	2.014468	COL1A1
hsa_circ_0044539	0.000852	3.267947	COL1A1
hsa_circ_0044542	0.016996	2.358792	COL1A1
hsa_circ_0044547	0.006894	2.410261	COL1A1
hsa_circ_0057410	0.039095	0.4518	COL3A1
hsa_circ_0089450	0.029781	3.464172	COL5A1
hsa_circ_0019953	0.001682	2.596717	COL17A1
hsa_circ_0065437	0.010592	0.429411	COL7A1

We hypothesized that COL1A1-related circRNAs play an important role in HCC LNM. Among COL1A1-related circRNAs, hsa_circ_0044539 exhibited the highest fold-change between the HCC LNM and HCC NLNM groups. Consequently, we selected hsa_circ_0044539 (hereafter referred to circ-0044539) for further research. According to circBase, circ-0044539 is at chr17:48266737-48273728 and has a spliced length of 1926 bp (Fig. 1D and Fig. S2A, B). To clarify the characteristics of circ-0044539, we designed circ-0044539-specific divergent primers to amplify the back-spliced products in HCC cell lines. The primer was characterized by a single peak of the solubility curve (Fig. S2C).

### Circ-0044539 promotes the proliferation, migration, and invasion of HCC cells in vitro

Circ-0044539 was expressed at higher levels in the MHCC97H, HCCLM3, and HCCLM6 cell lines (high invasive metastatic potential) than in the MHCC97L cell line (low invasive metastatic potential). Compared to normal hepatocyte L02 cell lines, these HCC cell lines showed higher circ-0044539 expression levels (Fig. 1E). Additionally, circ-0044539 exhibited resistance to RNase R treatment, whereas the linear isoform of COL1A1 did not (Fig. 1F). It also exhibited greater stability than COL1A1 mRNA (Fig. 1G). Significant enhancement of the proliferation, migration, and invasion capacity was observed with circ-0044539 overexpression, and these capacities were notably inhibited upon suppression of circ-0044539 (Fig. S2E and Fig. 1H–L). These findings illustrated the oncogenic role of circ-0044539 in HCC cells.

### Predicting the biological mechanism of circ-0044539 in HCC

**Fig. 2:**
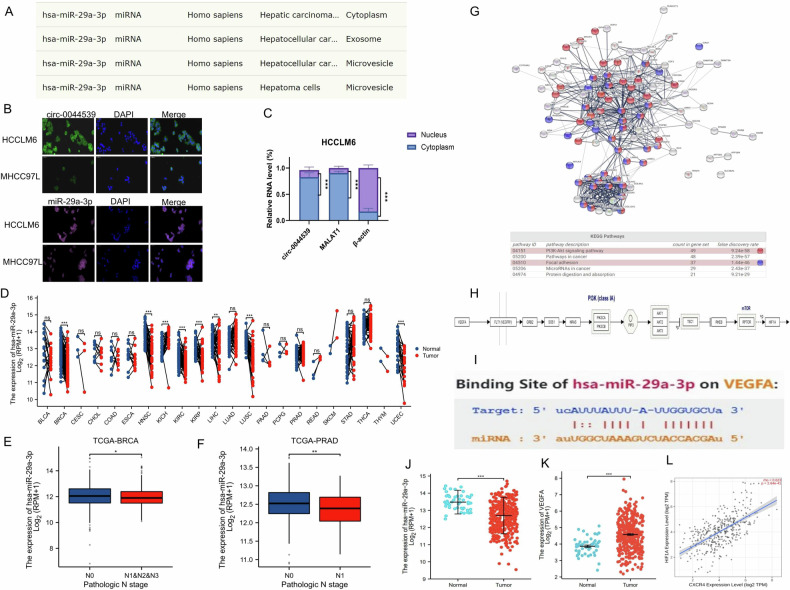
Expression analysis of miR-29a-3p and its downstream molecules. **A** The localization of miR-29a-3p in HCC, data from RNALocate (http://www.rna-society.org/rnalocate). **B** FISH showed the localization of circ-0044539 (green) and miR-29a-3p (pink) in HCC cell lines. Nuclei (DAPI) are shown in blue. Scale bar, 50 µm. **C** The location of circ-0044539 in HCC-LM6 cells was identified by subcellular fractionation assay and qRT-PCR (*n* = 3). MALAT1 and β-actin were used as the nuclear and cytoplasm controls, respectively. **D** The expression of miR-29a-3p in paired tumor and adjacent normal tissues in the indicated tumor types from the TCGA database, using Wilcoxon signed rank test. **E**, **F** miR-29a-3p expression in different N stages in BRCA (**E**) and PRAD (**F**). **G** STRING protein interaction analysis showed the downstream targets of miR-29a-3p. **H** Signaling pathway showed VEGFA-PI3K-Akt-mTOR-HIF-1α from the WikiPathway database. **I** The starBase database presented the binding site of miR-29a-3p on the VEGFA 3’UTR. **J**, **K** The expression levels of miR-29a-3p (**J**) and VEGFA (**K**) in HCC tissues and normal liver tissues. **L** The correlation between HIF-1α and CXCR4, data from the TCGA LIHC cohort was assessed with Spearman analysis. *p < 0.05, **p < 0.01, ***p < 0.001.

CircRNAs most commonly exert their function by sponging miRNAs [16]. Therefore, we utilized MRE analysis to forecast the potential target miRNAs of COL1A1-related circRNAs. These circRNAs have many binding sites with the miR-29 family, implying that the miR-29 family might serve as the main sponge miRNA of COL1A1-related circRNA. Notably, among these COL1A1-related circRNAs, circ-0044539 exhibited the highest number of binding sites with the miR-29 family, including miR-29a-3p, miR-29b-3p and miR-29b-3c (Table S3). The subcellular localization of molecules determines their potential molecular capabilities. As reported, miR-29a-3p is mainly localized in the cytoplasm and exosomes [17] (Fig. 2A), while miR-29b-3p and miR-29b-3c are mainly localized in the nucleus [18, 19]. Subcellular fractionation assay and RNA fluorescent in situ hybridization (FISH) experiments showed that circ-0044539 was predominantly localized in the cytoplasm of HCC cells (Fig. 2B, C). Thus, we focused on the circ-0044539-miR-29a-3p axis in our study.

As demonstrated in Fig. 2D, miR-29a-3p exhibited decreased expression levels in the majority of tumors compared with corresponding adjacent normal tissues. Furthermore, in BRCA and PRAD, miR-29a-3p expression was lower in the advanced N stage than in the N0 stage (Fig. 2E, F), indicating loss of miR-29a-3p might be associated with LNM events. As shown, downstream targets of miR-29a-3p were significantly enriched in “focal adhesion” and “PI3K-Akt” signals (Fig. 2G). Many LNM-related molecules are involved in the PI3K-Akt signaling pathway [20]. VEGFA plays a crucial role as a regulatory molecule of the PI3K-Akt-mTOR signaling pathway, and HIF-1α is a downstream molecule of the PI3K-Akt-mTOR signaling pathway (Fig. 2H). The starBase database revealed the specific binding site of miR-29a-3p on the VEGFA 3’UTR (Fig. 2I). Hypoxia has been validated as a typical characteristic of HCC [21]. Our previous studies have demonstrated that HIF-1α, VEGF, and CXCR4 are independent factors that influence HCC LNM [22, 23]. Studies have shown that hypoxia induces CXCR4 expression in chondrosarcoma, breast cancer, and colon cancer [24]. In the TCGA LIHC cohort, miR-29a-3p expression in tumors was lower than in normal liver tissues, while VEGFA expression was higher (Fig. 2J, K), and HIF-1α was positively associated with CXCR4 (Fig. 2L). Consequently, we postulated that circ-0044539 might function as a miR-29a-3p sponge, thereby enhancing HCC LNM ability through VEGFA. VEGFA activates PI3K-Akt-mTOR by binding to its receptor VEGFR, thereby increasing the expression of HIF-1α, which promotes the expression of CXCR4, a key factor in LNM.

### MiR-29a-3p inhibited the malignant behavior of HCC and reversed circ-0044539-induced tumor-promoting effects in vitro

To validate our hypothesis, miR-29a-3p knockdown and overexpression cell lines were constructed, and the expression of circ-0044539 remained unaffected despite the up- or downregulation of miR-29a-3p (Fig. S3A). MiR-29a-3p effectively suppressed cellular growth activity (Fig. S3B) and significantly decreased the migratory and invasive capabilities of the HCC cells (Fig. S3C–E). Downregulation of miR-29a-3p activated the PI3K-Akt-mTOR signaling pathway and increased the protein expression levels of HIF-1α and CXCR4 (Fig. S4B). In addition, VEGFA could attenuate these effects mediated by miR-29a-3p (Fig. S4A–F).

**Fig. 3:**
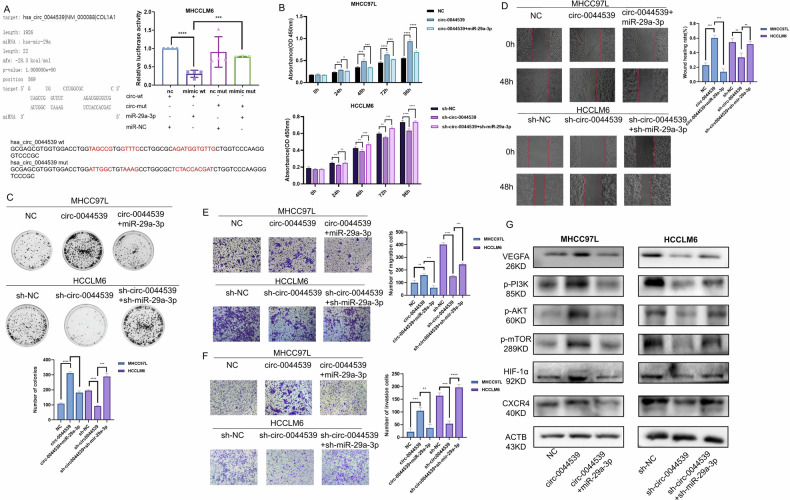
Circ-0044539 regulates the proliferation, migration, and invasion abilities of HCC cells through miR-29a-3p in vitro. **A** Dual-luciferase reporter gene assay showed a direct interaction between circ-0044539 and miR-29a-3p in HCCLM6 cells. **B**, **C** CCK-8 assays and plate colony formation experiments showed that miR-29a-3p reversed the circ-0044539-promoted proliferation abilities of HCC cells. **D**–**F** Wound healing assays (**D**) and transwell assays (**E**, **F**) showed that miR-29a-3p reversed the circ-0044539-promoted migration and invasion ability of HCC cells. **G** Western blot showed that circ-0044539 promotes the protein expression of downstream molecules that can be antagonized by miR-29a-3p. Data presented as means ± SD of three independent experiments. *p < 0.05, **p < 0.01, ***p < 0.001, ****p < 0.0001 (Student’s *t*-test).

A dual-luciferase reporter gene assay confirmed a direct interaction between circ-0044539 and miR-29a-3p (Fig. 3A). To further investigate the roles of the circ-0044539-miR-29a-3p axis in HCC progression, rescue assays were conducted. In addition, we found that circ-0044539 might affect miR-29a-3p expression level in HCC cell lines in relation to culture condition, cell density, and cell doubling time (data not shown). The rescue assay results demonstrated that the promotive effects of circ-0044539 overexpression on proliferation, migration, and invasion abilities could be reversed by miR-29a-3p and that the suppressive effects mediated by circ-0044539 knockdown could be reversed by inhibiting miR-29a-3p (Fig. 3B–G). As demonstrated in Fig. 3G, circ-0044539 increased the phosphorylation level of PI3K-Akt-mTOR signaling pathway, and increased the protein expression of HIF-1α and CXCR4, and these effects were reversed by miR-29a-3p. Taken together, these results indicated that miR-29a-3p exhibits anti-carcinogenic effects and partially mediates the oncogenic function of circ-0044539 through direct binding in HCC.

### Effects of the circ-0044539-miR-29a-3p axis on the growth and LNM of HCC in vivo

**Fig. 4:**
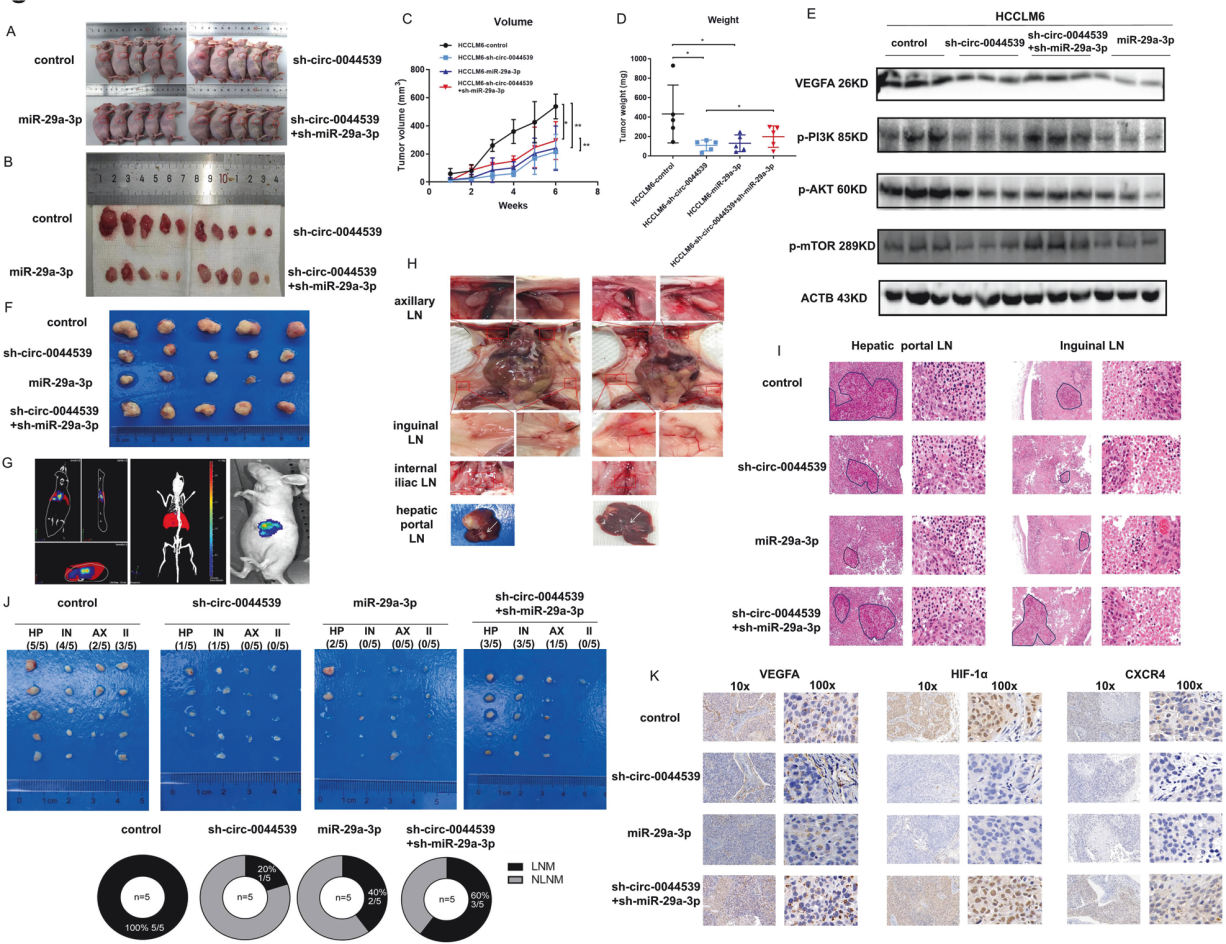
The effects of circ-0044539-miR-29a-3p on the growth and LNM of HCC in vivo. **A**–**D** Presentation of subcutaneous tumors in nude mice (**A**, **B**) with corresponding tumor volumes (**C**) and tumor weights (**D**) (*n* = 5). The HCCLM6 cell line is utilized as the source of human HCC cells for animal experiments. **E** Western blot analysis showed the protein expression level of the VEGFA-PI3K-Akt-mTOR pathway in different groups of subcutaneous tumors. **F** Photograph of liver orthotopic tumors (*n* = 5) of HCCLM6. **G** Representative BLI photograph of liver orthotopic tumors. **H** Anatomical presentation of representative LNs. **I** Representative HE image of LNM. **J** Photograph of LNs separated from liver orthotopically implanted nude mice (*n* = 5). The LNM rates were calculated. HP hepatic portal LN, IN inguinal LN, AX axillary LN, II internal iliac LN. **K** Representative IHC images of VEGFA, HIF-1α, and CXCR4 in liver orthotopic tumors. Data presented as means ± SD of three independent experiments. *p < 0.05, **p < 0.01 (Student’s *t*-test).

Both a subcutaneous xenograft model and a liver orthotopic implantation nude mouse model were established with four groups of HCCLM6 cells (control, sh-circ-0044539, miR-29a-3p, sh-circ-0044539+sh-miR-29a-3p). The results of subcutaneous xenograft demonstrated that the sh-circ-0044539 groups and the miR-29a-3p group exhibited slower tumor growth than the NC group, whereas the sh-circ-0044539+sh miR-29a-3p group displayed faster tumor growth than the sh-circ-0044539 group (Fig. 4A–D). Western blotting results showed that the circ-0044539-miR-29a-3p axis regulated the VEGFA-PI3K-Akt-mTOR pathway (Fig. 4E).

The results of the liver orthotopic implantation model were consistent with the findings of the subcutaneous xenograft experiment (Fig. 4F). Furthermore, LNs were separated and stained by HE to confirm LNM events (Fig. 4G–I). The incidence of LNM was 20% (1/5) in the HCCLM6-sh-circ-0044539 group, which was significantly lower than that in the HCCLM6-control group (100%, 5/5). The incidence of LNM in the HCCLM6-miR-29a-3p group was 40% (2 of 5), and that in the HCCLM6-sh-circ-0044539+sh-miR-29a-3p group was 60% (3 of 5) (Fig. 4J). HCCLM6-control and HCCLM6-sh-circ-0044539+sh-miR-29a-3p groups had a higher density of tumor cells in LNs than HCCLM6-sh-circ-0044539 group. Moreover, impaired VEGFA, HIF-1α, and CXCR4 expression were observed in the sh-circ-0044539 and miR-29a-3p groups, compared with the HCCLM6-control group. The tumors of the HCCLM6-sh-circ-0044539+sh-miR-29a-3p group had higher levels of VEGFA, HIF-1α, and CXCR4 than those of the HCCLM6-sh-circ-0044539 group (Fig. 4K). In vivo, assays demonstrated that knockdown of circ-0044539 or overexpression of miR-29a-3p dramatically inhibited the oncogenic and LNM potential of HCC cells, and knockdown of circ-0044539 affected HCC LNM in a miR-29a-3p-dependent manner.

### Circ-0044539 promotes exosome secretion and exosomal miR-29a-3p enrichment in a HIF-1α-dependent manner

According to the functional enrichment analysis results, LNM-related circRNAs might be involved in vesicle secretion (Fig. 1C). Exosomes are a significant category of biological vehicles that can be secreted by diverse cell types and have the potential to alter the behavior of recipient cells.

**Fig. 5:**
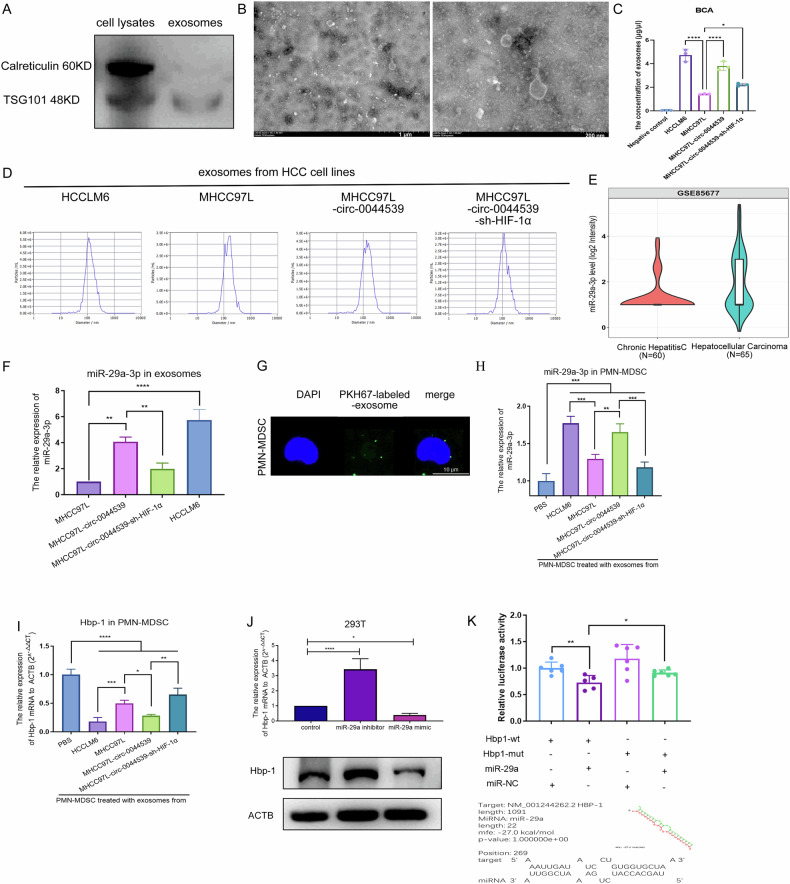
Circ-0044539 increased HCC exosome and exosomal miR-29a-3p levels in a HIF-1α-dependent manner. **A** Western blot assay of the exosomal marker TSG101 and endoplasmic reticulum marker Calreticulin. Exosomes were extracted from MHCC97L. **B** TEM of HCC-derived exosomes. Scale bar, 1 µm, 200 nm. **C** The quantity of exosomes was determined by BCA assay. **D** NanoSight particle tracking analysis showing the particle size of HCC-derived exosomes. **E** Expression of miR-29a-3p in exosomes from chronic and HCC patients, data from GSE85677. **F** The relative expression of miR-29a-3p to U6 in HCC exosomes (500 ng RNA) was detected through qRT-PCR. **G** PMN-MDSC uptook PKH67-labeled HCC-exosomes. **H**, **I** After treatment with different groups of HCC exosomes (2 µg/10^6^ PMN-MDSCs), miR-29a-3p (**H**) and Hbp-1 mRNA expression (**I**) in PMN-MDSCs were detected. **J** After transfection with the miR-29a-3p mimic or inhibitor, the Hbp-1 mRNA and protein levels of HEK293T cells were detected. **K** Luciferase reporter activity was analyzed in HEK293T cells after treatment with miR-29a-3p mimics and miR-NC. The wild-type (WT) or mutant (MT) MRE of the Hbp-1 3’-UTR was constructed and transfected into HEK293T cells. Data presented as means ± SD of three independent experiments. *p < 0.05, **p < 0.01, ***p < 0.001, ****p < 0.0001 (Student’s *t*-test).

Given the crucial role of exosomes in mediating the interaction between cancer cells and the microenvironment of the target organ, we extracted exosomes derived from HCC. The typical exosome biomarker Tsg101 was present, while Calreticulin was absent in the extract (Fig. 5A). Transmission electron microscopy (TEM) showed the typical morphology of vesicular body-like structures (Fig. 5B). NTA demonstrated that the size of the samples (Fig. 5C). All these characteristics confirmed that we successfully isolated and purified exosomes from HCC. The NTA assays and BCA assays confirmed that the number of exosomes was regulated by circ-0044539 (Fig. 5C, D).

According to the data presented, miR-29a-3p is located in the cytoplasm and exosomes (Fig. 2A). Interestingly, miR-29a-3p is downregulated in HCC tumors compared to para-carcinoma tissues (Fig. 2J), but has a higher expression level in exosomes derived from HCC patient serum than in those from healthy donors [25]. This finding is supported by the results of GSE85677 analysis, which demonstrated that miR-29a-3p was upregulated in serum exosomes from HCC patients compared to those from chronic hepatitis patients (Fig. 5E). When miR-29a-3p expression was low in HCC cells, it was high in HCC exosomes. We reasoned that HCC cells release a large number of exosomes containing miR-29a-3p, which leads to a low expression level of miR-29a-3p in the cell.

Reportedly, exosome secretion is an elaborate process regulated by specific factors, such as HIF-1α and oxygen conditions [26]. As demonstrated previously, circ-0044539 upregulated HIF-1α in HCC. Therefore, we hypothesized that under hypoxic conditions, HIF-1α promotes exosome secretion. Based on the result that the circ-0044539-HIF-1α axis regulates exosome production in HCC (Fig. 5C and Table 2), we further investigated the expression of miR-29a-3p in these exosomes and found that miR-29a-3p was enriched in exosomes derived from MHCC97L-circ-0044539 compared with those derived from MHCC97L. Knockdown of HIF-1α not only decreased the number of exosomes from MHCC97L-circ-0044539 but also decreased the exosomal miR-29a-3p expression level (Fig. 5F). In conclusion, the circ-0044539-HIF-1α axis regulates the number of exosomes and the enrichment of miR-29a-3p in exosomes. However, it is not clear how circ-0044539 or HIF-1α mediates the generation and packaging of miR-29a-3p into exosomes.

**Table 2:** The NTA analysis of the particle distribution of exosomes derived from HCC cell lines.

Exosomes from	Average diameter (nm)	Concentration (particles/mL)
HCCLM6	139.2	7.2 × 10^10^
MHCC97L	160.0	7.0 × 10^9^
MHCC97L-circ-0044539	153.6	5.3 × 10^10^
MHCC97L-circ-0044539-sh-HIF-1α	133.0	3.2 × 10^10^

### Exosomal miR-29a-3p regulates the Hbp-1 expression in PMN-MDSC

LN is an immuno-positive organ, with multiple infiltrating immune cells. Exosomal-miR-29a-3p has been found to be an immune cell regulator [27]. We screened research about the effects of exosomal miR-29a-3p on immune-related cells to find out the mechanism by which exosomes derived from HCC cells influence the LN microenvironment. We identified 11 relevant reports, and 9 potential miR-29a-3p targets were found (Table 3). Among these, we are interested in Hbp-1, as Hbp-1 is regulated by hypoxia-induced exosomes and downregulated Hbp-1 promotes MDSC immunosuppressive ability [8]. MDSCs play an important role in the tumor environment, but there have only been a few studies on the role of MDSCs in LNs, despite that MDSCs are more highly enriched in LNs than in solid tumor tissues [4].

**Table 3:** Downstream gene and pathway of exosomal-miR-29a-3p.

Research (year)	Exosomes from:	Exosome receiver:	miR-29a-3p target gene:	Downstream gene/pathway
Cai et al. [46]	Oral squamous cell carcinoma	Macrophage	SOCS1	p-STAT6
Bai et al. [47]	Placental	Monocytes	PTEN	PD-L1
Zhou et al. [48]	TAM	CD4 + T	STAT3	Treg/Th17 rate; IL-6, TNFa, IL-10 in T cell
Lu et al. [49]	TAM	Ovarian cancer	FOXO3	FOXO3-AKT-GSK3β-PD-L1
Zhu et al. [50]	G-MDSC	Th1	T-bet, STAT3	Th1
Fabbri et al. [51]	Tumor cell	Macrophage	TLR8	NF-kb-TNFa-IL-6
Sun et al. [52]	Pancreatic β cell	Monocyte, macrophage	TRAF3	Inflammation
Wang et al. [53]	Bone marrow macrophage	Monocyte, macrophage	MCL-1	Inflammation
Guo et al. [8]	Hypoxia-induced glioma cells	MDSC	Hbp1	Functional MDSC expansion
Liu et al. [54]	Macrophages reside within adipose tissue	Adipocytes, myocytes, and hepatocytes	PPAR-δ	Insulin resistance
Chen et al. [55]	Lung epithelial cells	Macrophages		PI3K-AKT, MAPK

To directly investigate the mechanism of exosomal miR-29a-3p in MDSCs, we sorted PMN-MDSC for further research, as PMN-MDSCs represent the most abundant population of MDSCs [28]. Manual gating of these immune clusters is shown in Fig. S5A. Then these PMN-MDSCs were cultured with exosomes isolated from 4 different groups of HCC cell lines (HCCLM6, MHCC97L, MHCC97L-circ-00445309, MHCC97L-circ-00445309-sh-HIF-1α). Immunofluorescence imaging showed the presence of PKH67-labeled exosomes in PMN-MDSC (Fig. 5G). To investigate the effects of HCC-derived exosomes on PMN-MDSCs, we examined the expression of miR-29a-3p and Hbp-1 at both mRNA and protein levels after co-culture. As shown in Fig. 5H, I, the MDSC1 (PMN-MDSC treated with HCCLM6-exosomes) and MDSC3 (PMN-MDSC treated with MHCC97L-circ-0044539-exosomes) group exhibited higher miR-29a-3p and lower Hbp-1 mRNA levels than the MDSC2 group (PMN-MDSC treated with MHCC97L-exosomes). MDSC4 (PMN-MDSC treated with MHCC97L-circ-0044539-sh-HIF-1α-exosomes) exhibited lower levels of miR-29a-3p and higher levels of Hbp-1 mRNA than MDSC3 (PMN-MDSC treated with MHCC97L-circ-0044539-exosomes). The expression of Hbp-1 in PMN-MDSCs is regulated by HCC-derived exosomes, via the circ-0044539-HIF-1α mechanism.

Then, 293 T cells were used to investigate whether miR-29a-3p affects Hbp-1 expression. Hbp-1 expression was significantly decreased after the forced expression of miR-29a-3p and was increased after the knockdown of miR-29a-3p (Fig. 5J). Luciferase activity assay showed that compared with the negative control, miR-29a-3p mimics significantly reduced the luciferase reporter activity of wild-type Hbp-1, and had less effect on mutated Hbp-1 (Fig. 5K). These results confirmed that the transfer of miR-29a-3p from HCC exosomes to PMN-MDSCs causes downregulation of Hbp-1.

### HCC exosomes regulate the immunosuppressive feature of PMN-MDSCs

**Fig. 6:**
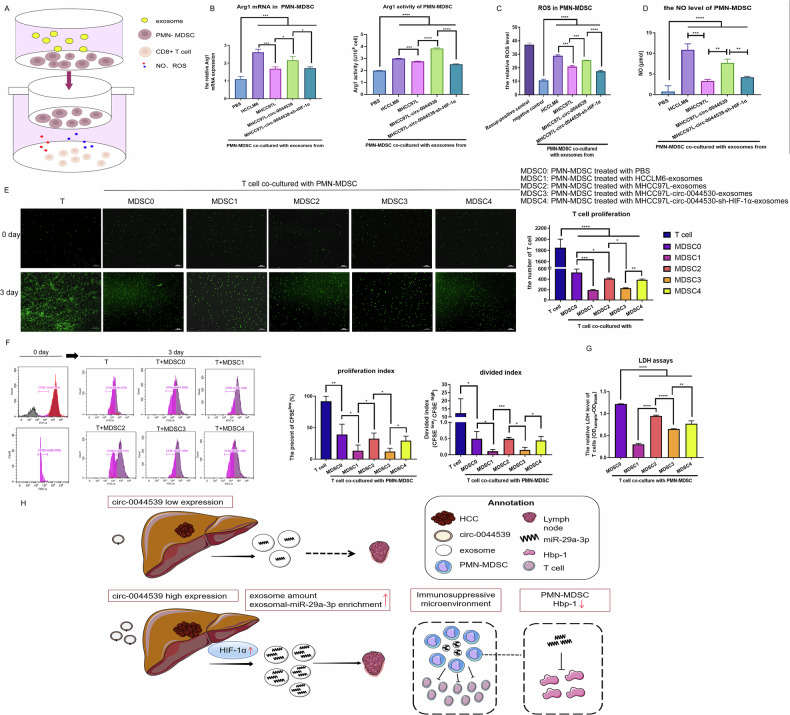
Increased immunosuppression activity of PMN-MDSCs following treatment with exosomes derived from HCC. **A** Experimental schematic diagram of PMN-MDSCs, sorted from HCC patients and then co-cultured with HCC exosomes, cultured with CD3 + CD8 + T cells isolated from healthy donors to evaluate PMN-MDSC function. **B** The Arginase mRNA expression level and Arginase activity in PMN-MDSCs with the indicated HCC-exosome treatments were assessed. **C**, **D** The ROS and NO levels in PMN-MDSCs with indicated treatments were detected. **E**, **F** The CFSE staining assay showed T-cell proliferation. Representative fluorescence diagram (**E**) and flow cytometric data (**F**) are shown. The percentage of the CFSE low population represents the proportion of proliferating CD3 + CD8 + T cells. The proliferation index and the divided index were calculated. **G** LDH release assays of T cells co-cultured with the indicated PMN-MDSCs were detected. **H** A schematic illustration of the molecular mechanism through which circ-0044539-derived exosomal miR-29a-3p regulates PMN-MDSC immunosuppression ability. Data presented as means ± SD of three independent experiments. *p < 0.05, **p < 0.01, ***p < 0.001, ****p < 0.0001 (Student’s *t*-test).

Then, we assessed the immunosuppressive capacity of PMN-MDSCs by measuring the levels of ROS, NO, and Arg1 activity (Fig. 6A). As observed, the highest levels of Arg1 mRNA expression and its activation level, as well as the levels of ROS and NO production, were evident in MDSC1. MDSC2 and MDSC4 showed lower levels of Arg1, ROS, and NO than MDSC3 (Fig. 6B–D).

The CD8 + T cells were co-cultured with PMN-MDSCs pre-treated with exosomes from HCC cells and then were detected through LDH release and CFSE assays. T-cell cytotoxicity and proliferation were significantly decreased following PMN-MDSC treatment (Fig. 6E–G). These results also confirmed that circ-0044539 induced increases in ROS level, NO level, and Arg1 activation in PMN-MDSCs, and subsequently decreased T-cell proliferation and cytotoxicity, which were attenuated by HIF-1α knockdown (Fig. 6E–H).

### Immune microenvironment changes occur in LNs under exosome treatment

Based on these data, we reasoned that rapid cell proliferation facilitated by circ-0044539 leads to localized hypoxia in HCC, which in turn triggers the packaging of miR-29a-3p into exosomes and the secretion of exosomes into lymphatic vessels to influence PMN-MDSCs in LNs. The pre-metastatic LN niche formed before tumor cell arrival plays a vital role in sustaining metastatic tumor cell growth. We further validated our hypothesis in the Hu-NSG, a mouse model with a reconstituted human immune system [29]. Human CD45+ cells harvested from the blood of Hu-NSG mice accounted for 25.52% of all CD45+ cells, confirming the successful construction of Hu-NSG mice (Fig. S6A).

In our study, a footpad inoculation model of HCC was established to investigate LNM patterns. After footpad inoculation, methylene blue dye moved from the footpad to the popliteal LN through lymphatic vessels (Fig. S6C). We injected PKH67-labeled exosomes into the footpad of Hu-NSG and harvested the popliteal LN after 24 hours (Fig. S6D, E). More interestingly, exosomes (labeled with PKH67) selected the target cell rather than being distributed randomly. The sensitivity of PKH67 dye for detecting exosome migration and the loss of exosome amount in the process of dying were not satisfied. Therefore, we opted for iRFP682, which has stable luminescent properties and good biological properties, as a more suitable alternative [30]. The iRFP682-labeled exosomes of HCCLM6-control and HCCLM6-circ-0044539 were extracted.

**Fig. 7:**
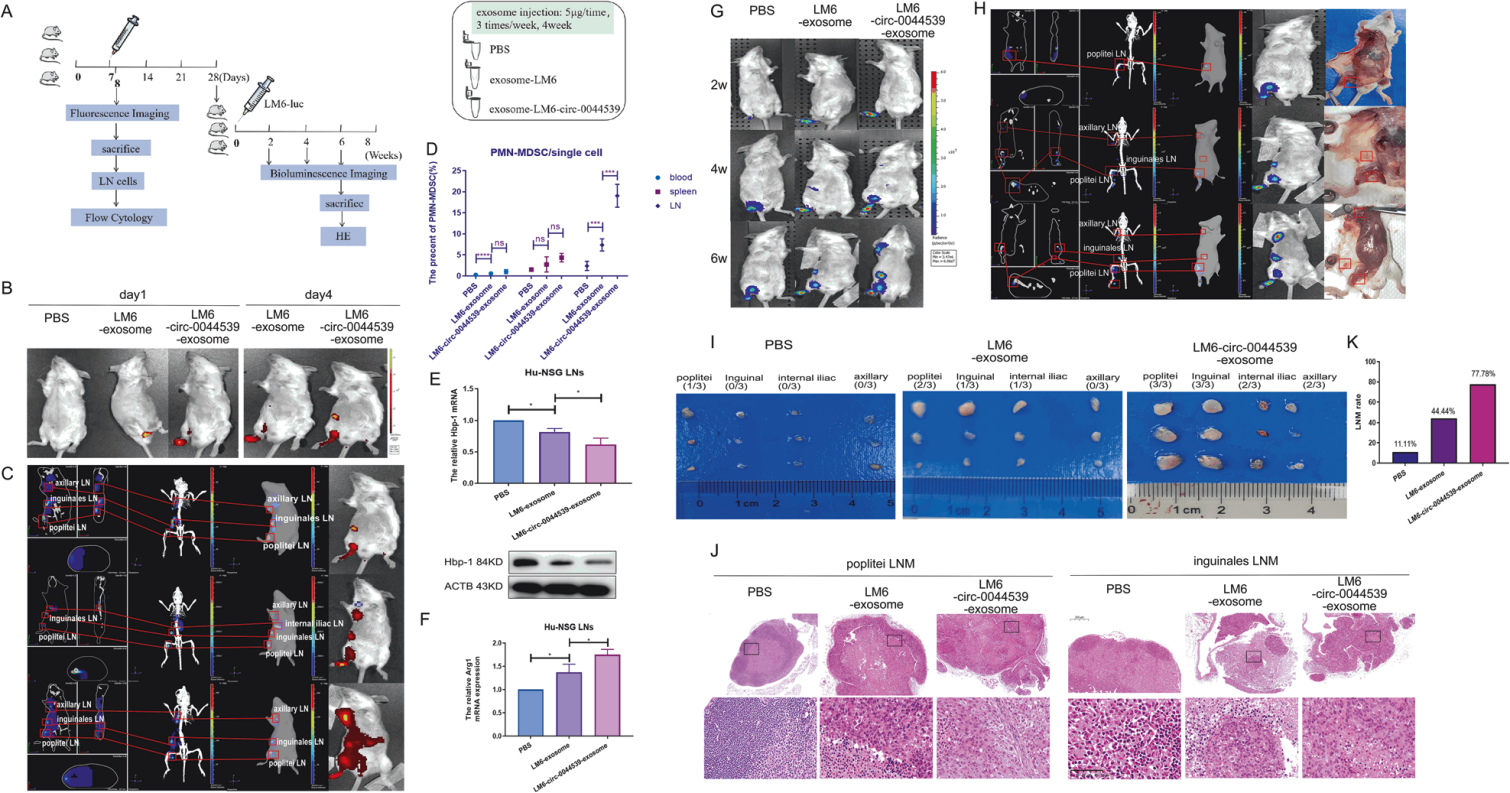
HCC-derived exosomes reconstructed the LN microenvironment and promoted LNM in Hu-NSG. **A** Schematic of the proposed Hu-NSG model. Hu-NSG was treated with PBS or HCC-exosome to construct a pre-metastatic mice model, and then Hu-NSG was injected with HCCLM6-luciferase to evaluate the effect of pre-metastatic environmental change on HCC LNM. **B**, **C** iRFP682-labeled exosomes derived from HCCLM6 cells were injected into the footpad of Hu-NSG mice. After the HCC-exosome was transferred into recipient Hu-NSG, fluorescence signals in Hu-NSG were observed by an imaging system (**B**), as well as a 3D fluorescence imaging system (**C**). **D** The percentage of PMN-MDSCs in leukocytes, spleen, and popliteal LNs was determined. **E**, **F** The expression of Hbp-1 (**E**) and Arg1 (**F**) in LN cells from Hu-NSG mice after footpad inoculation was detected. The value from the HCC-exosomes group was normalized to the PBS group (*N* = 3/group). **G** Serial BLI images at different time points of Hu-NSG. **H** Typical BLI 3D image of Hu-NSG with LNM. **I** Photograph of LN separated from Hu-NSG. **J** Representative HE image of LNM. **K** The LNM rate was calculated. Data presented as means ± SD of three independent experiments. *p < 0.05, ***p < 0.001, ****p < 0.0001 (Student’s *t*-test).

After injecting PBS, HCCLM6-control-exosomes or HCCLM6-circ-0044539-exosomes into the right footpad of the Hu-NSG mice three times in one week, the migration process of exosomes was observed by fluorescence imaging (Fig. 7A–C). We assessed the accumulation of PMN-MDSCs in the blood, spleen, and LNs, one day after the final exosome injection in the first week. Significantly more PMN-MDSCs were detected in Hu-NSG blood and LNs after exosomes pre-treatment than after PBS pre-treatment (Fig. 7D). A higher PMN-MDSC proportion was observed in the LNs of the HCCLM6-circ-0044539-exosomes group than in those of the HCCLM6-control-exosomes group. Although statistical significance was not achieved for the PMN-MDSC proportion change in the spleen of Hu-NSG, a trend toward significance was observed between the three groups (Fig. 7D). These results showed that HCC-exosomes might primarily function within LN structures, forested an immunosuppressive microenvironment through PMN-MDSCs, and then influence the peripheral blood or spleen. Further experiments showed that both HCCLM6-control-exosomes and HCCLM6-circ-0044539-exosomes decreased Hbp-1 protein and mRNA and Arg1 mRNA in LN cells (Fig. 7E, F). HCCLM6-circ-0044539-exosomes decreased the levels of these indicators to a greater extent than HCCLM6-control-exosomes (Fig. 7E, F).

HCCLM6-luciferase cells were injected into the footpad of all three Hu-NSG groups, which were pre-treated with PBS or exosomes three times/week for four weeks. After injection, from week 2 to week 6, the metastasis patterns of LNs in Hu-NSG were investigated through in vivo BLI (Fig. 7G, H). All LNs were collected at week 6 (Fig. 7I). HE examination further confirmed the presence of metastatic HCCLM6 cells in the LNs (Fig. 7J). Likewise, LNM number and degree were significantly increased in the HCCLM6-circ-0044539-exosomes pre-treatment group compared with the HCCLM6-control-exosomes pre-treatment group (Fig. 7J–K). Moreover, mice in the PBS group had only small metastatic lesions in popliteal LN.

Notably, the frequency of LNM increased from 11.11% (1/12) in the PBS group, to 44.44% (4/12) in the HCCLM6-control-exosomes group, and to 77.78% (10/12) in the HCCLM6-circ-0044539-exosomes group at week 6 (Fig. 7K). These results confirmed that circ-0044539 in HCCLM6 created an immunosuppressive LN microenvironment through exosomes and led to increased LNM rates in the HCC model.

### Serum circ-0044539 and plasma exosomal miR-29a-3p were specific biomarkers for HCC LNM

**Fig. 8:**
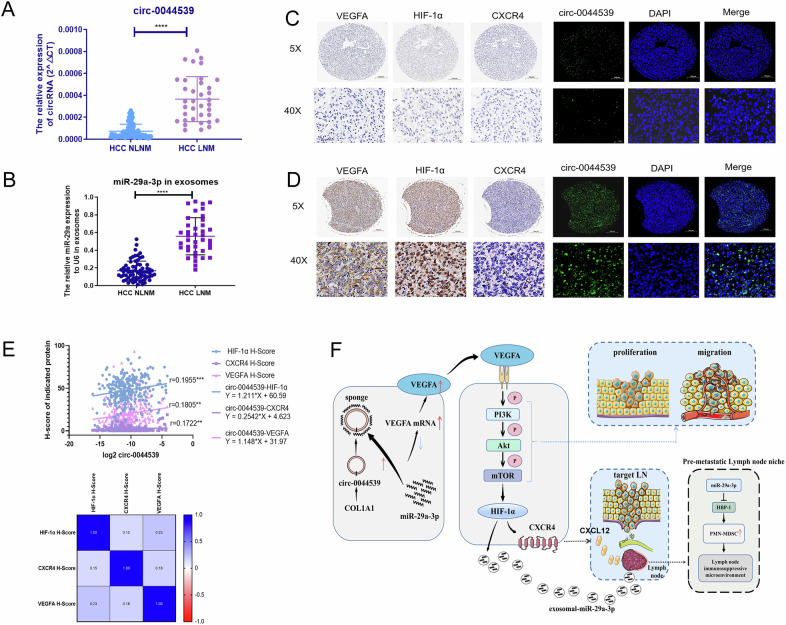
Circ-0044539 serves as a novel biomarker for HCC patients with LNM. **A** The expression levels of circ-0044539 in serum samples from HCC patients with LNM (*n* = 36) and without LNM (*n* = 88) were determined by qRT-PCR. **B** The expression levels of miR-29a-3p expression in plasma exosomes from HCC patients with LNM (*n* = 39) and without LNM (*n* = 69). **C**, **D** Representative images of FISH and IHC analysis of circ-0044539, VEGFA, HIF-1α, and CXCR4 in HCC patients without (**C**) or with (**D**) LNM. **E** The correlation relationship between the circ-0044539-VEGFA-HIF-1α-CXCR4 axis. **F** Schematic of the proposed model. A Spearman correlation was used for statistical analysis. Tests between the two groups used a two-tailed unpaired Student’s *t*-test. **p < 0.01, ****p < 0.0001.

We evaluated the serum concentration of circ-0044539 in 124 HCC patients (LNM:36, NLNM:88), and noted that serum circ-0044539 was markedly higher in the LNM group than in the NLNM group (Fig. 8A). Moreover, we found that plasma exosomal miR-29a-3p levels were notably elevated in HCC patients with LNM compared to those without LNM (Fig. 8B). These findings suggest that circulating circ-0044539 and exosomal miR-29a-3p may serve as potential biomarkers for the diagnosis of HCC LNM.

### Intratumoral circ-0044539 and miR-29a-3p are correlated with LNM in HCC patients

Then, we performed FISH and IHC staining of a TMA containing 295 HCC tumors. Typical FISH and IHC images of circ-0044539, VEGFA, HIF-1α, and CXCR4 are presented in Fig. 8C, D. The circ-0044539-VEGFA-HIF-1α-CXCR4 axis showed minimal intensity in tumor tissues from HCC patients without LNM and obvious upregulation of expression in the tumor tissues of HCC patients with LNM. We investigated the correlation between circ-0044539 expression and the expression of VEGFA, HIF-1α, and CXCR4 in HCC tissues. We found that circ-0044539 expression was positively correlated with VEGFA (*r* = 0.1722, *p* = 0.003), HIF-1α (*r* = 0.1955, *p* = 0.0008) and CXCR4 (*r* = 0.1805, *p* = 0.0019; Fig. 8E) expression. Circ-0044539 expression levels are associated with Tumor size (*p* < 0.01), and BCLC stage (*p* < 0.01) (Table 4).

**Table 4:** The association between circ-0044539 expression and clinical factors in HCC patients.

Variable	No. of patients	n/All	circ-0044539		*p*-value
			Low *n* = 148	High *n* = 147	
Age					
≤60	148	0.502	69	79	0.221
>60	147	0.498	79	68	
Gender					
Female	75	0.254	35	40	0.482
Male	220	0.746	113	107	
HBsAg					
Negative	71	0.241	33	38	0.475
Positive	224	0.759	115	109	
HCV-Ab					
Negative	288	0.976	145	143	0.695
Positive	7	0.024	3	4	
AFP, ng/mL					
≥20	99	0.336	52	47	0.565
>20	196	0.664	96	100	
ALT, U/L					
≤40	178	0.603	87	91	0.584
>40	117	0.397	61	56	
Liver cirrhosis					
No	41	0.139	23	18	0.413
Yes	254	0.861	125	129	
Child–Pugh score					
A	291	0.986	147	144	0.311
B	4	0.014	1	3	
Tumor differentiation					
I–II	209	0.708	102	107	0.465
III–IV	86	0.292	46	40	
Tumor size, cm					
≤5	202	0.685	113	89	0.004**
>5	93	0.315	35	58	
Tumor number					
Single	223	0.756	114	109	0.565
Multiple	72	0.244	34	38	
Tumor encapsulation					
Complete	152	0.515	78	74	0.685
None	143	0.485	70	73	
Vascular invasion					
No	233	0.790	115	118	0.588
Yes	62	0.210	33	29	
BCLC stage					
0–A	187	0.634	106	81	0.003**
B–C	108	0.366	42	66	

**p < 0.01.

## Discussion

The 5-year overall survival rate of HCC patients remains unsatisfactory due to relapse with distant metastasis and resistance to anti-tumor agents, such as ICIs. For many solid malignancies, LNM represents a harbinger of distant metastatic disease and is also an important prognostic factor [31]. Previous studies have indicated that LNM patterns in tumors are notably complex, due to special immune response patterns in LNs [32]. The ‘seed and soil’ theory proposes that HCC LNM initiation depends on the synergy of HCC cells (seed) and LN microenvironment (soil). The primary tumor prepares the tumor-draining lymph nodes (TDLNs) for metastatic tumor cell accommodation. Exosome transfer might be the key mechanism through which primary tumors modulate distant TDLN microenvironments. However, this phenomenon remains unclear in HCC. Accordingly, by elucidating the underlying mechanisms involved in the distant regulation progress between HCC cells and the LN microenvironment, we could intervene in the progression of LNM.

Studies have demonstrated that dysregulated circRNAs play an important role in the occurrence and progression of various cancers [33]. In this study, we found that HCC LNM-related circ-00044539 affects HCC cell proliferation, migration, and invasion abilities in vitro and in vivo. Furthermore, we delineated the cellular signaling pathway of circ-0044539: miR-29a-3p-VEGFA-HIF-1α-CXCR4. Previous studies showed that VEGFA could promote angiogenesis in HCC and lead to dysregulation of its downstream PI3k-AKT-mTOR pathway [34]. High VEGFA expression in HCC was associated with poorer survival and poorer predicted ICB response. As reported, PPARγ supported the development of an MDSC-enriched and T-cell-dysfunctional TME through VEGFA trans-activation [35]. In the local tumor site and the TDLNs, MDSCs decomposed arginine and tryptophan, which are necessary for T-cell proliferation, eventually leading to the T-cell arrest in the G0 phase, and produce NO to inhibit the JAK3/STAT5 signaling pathway to reduce the expression of MHC II molecules, finally resulting in the apoptosis of T cells [36]. Studying the regulatory role and mechanism of HCC cells on MDSCs and then specifically downregulating the immune-suppressive effects of MDSCs could not only improve the anti-tumor immune function in LNs and inhibit the occurrence of LNM but could also provide active anti-tumor immune cells to enhance the effect of immunotherapy.

Tumor cells, stromal cells, and activated immune cells can produce a series of molecules that affect the amplification and immunosuppression of MDSCs. These molecules could be divided into two groups: 1. Molecules that mediate the expansion of MDSCs, including granulocyte-macrophage colony-stimulating factor (GM-CSF), stem cell factor (SCF), VEGF, etc. 2. Molecules that mediate the activation of MDSCs, including IFN-γ, IL-1β, IL-4, IL-6, IL-13, TNF and HMG1-1, etc [37]. Hbp-1 belongs to the HMG transcription factor family and can bind to specific sequences (the TTCATTCATTCA binding site) in the locus control region (LCR) of hCD2, maintain open chromatin conformation and direct gene expression [38], regulate the expression of a series of cell cycle-related genes (such as CDKN2A, CDKN1A and CCND1) and inhibit cell proliferation in many tumors [39]. Silencing Hbp-1 promotes Arg1 and NO levels in MDSCs [8], which hinders the proliferation of T cells and leads to T-cells unresponsiveness. Thus, T cells are unable to clear circulating tumor cells and locally implanted tumor cells. Our study further explored that HCC cells downregulated Hbp-1 in PMN-MDSCs through exosomal miR-29a-3p, and confirmed the role of HCC-exosomes in regulating PMN-MDSC function. This study indicates that dysregulated genes in HCC weaken the anti-tumor effect of the immune system via exosomes.

In line with previous studies, our results of animal experiments showed that activated PMN-MDSCs in LNs facilitate LNM. Regional LNs are the primary site of acquired immunity and the main defense line against tumors [40]. However, in TDLNs, due to the inhibitive immune microenvironment and continuous tumor antigen stimulation, CD8 + T cells eventually differentiate into terminally exhausted T cells, which are difficult to reactivate [41]. Heeren found that LNs with metastatic lesions have more immune-suppressive features than those without metastatic lesions [42]. This immunosuppressive state of LNs is conducive to tumor cell spread and even contributes to the development and metastasis of the tumor. Recent studies have revealed the importance of immune-active LNs in immune therapy [43]. Nanoparticles containing cGAMP could enhance STING signal transduction in sentinel LNs and transform immune-suppressive microenvironments into immune-active environments [44]. Studies have found that isolating tumor-draining lymph node-derived tumor-specific memory T cells (TdLN-TTSM), expanding TdLN-TTSM in vitro, and then infusing TdLN-TTSM back into the patient, could enhance the adoptive T-cell therapy effect against tumors, and inhibit tumor recurrence and metastasis after surgery [45]. As protecting TDLNs could be beneficial for clinical treatment, exploring the mechanism of pre-metastatic LN microenvironment formation and reversing the immunosuppressive state of LNs, will be a promising new strategy for HCC treatment. The lymphatic drainage areas of different primary tumors have varying microenvironment patterns, which are unclear in HCC. This study is the first attempt to elucidate the molecular mechanisms that govern the microenvironment of LNs prior to HCC metastasis.

Overall, our study demonstrated that circ-0044539 could affect both HCC cells and the LN microenvironment to promote HCC LNM (Fig. 8F). Circ-0044539 enhanced the proliferation, migration, and invasion capabilities of HCC cells via miR-29a-3p. Within HCC cells, circ-0044539 upregulated the expression of CXCR4 and promoted the release of extracellular exosomes enriched with miR-29a-3p via a HIF-1α-dependent pathway. These exosomes affected the expression of Hbp-1 in PMN-MDSCs, thereby enhancing the immunosuppressive function of PMN-MDSCs. In vivo, experiments clearly showed that exosomes from HCC cells with high expression of circ-0044539 target LNs and form a pre-metastatic LN microenvironment that promotes HCC LNM. Analysis of this HCC cohort unveiled that the expression of circ-0044539 in tissues and serum may serve as a potential biomarker for HCC LNM. These results suggested that high expression of circ-0044539 acquired by incipient HCC cells confers not only a selective survival advantage but also the tendency to home to selected organs: LNs, which might lead to distant spread and immunosuppression.

### Supplementary information

Original Data File

Supplementary Materials

## Data Availability

All data used to support the findings of this study are available from the corresponding author upon request.
