# Supplementary material for: Circ-0044539 promotes lymph node metastasis of hepatocellular carcinoma through exosomal-miR-29a-3p
Source: Cell Death Dis. 2024 Aug 27;15(8):630. doi: 10.1038/s41419-024-07004-x (PMC11349895; doi:10.1038/s41419-024-07004-x)

Unprocessed gels of Figure 3G

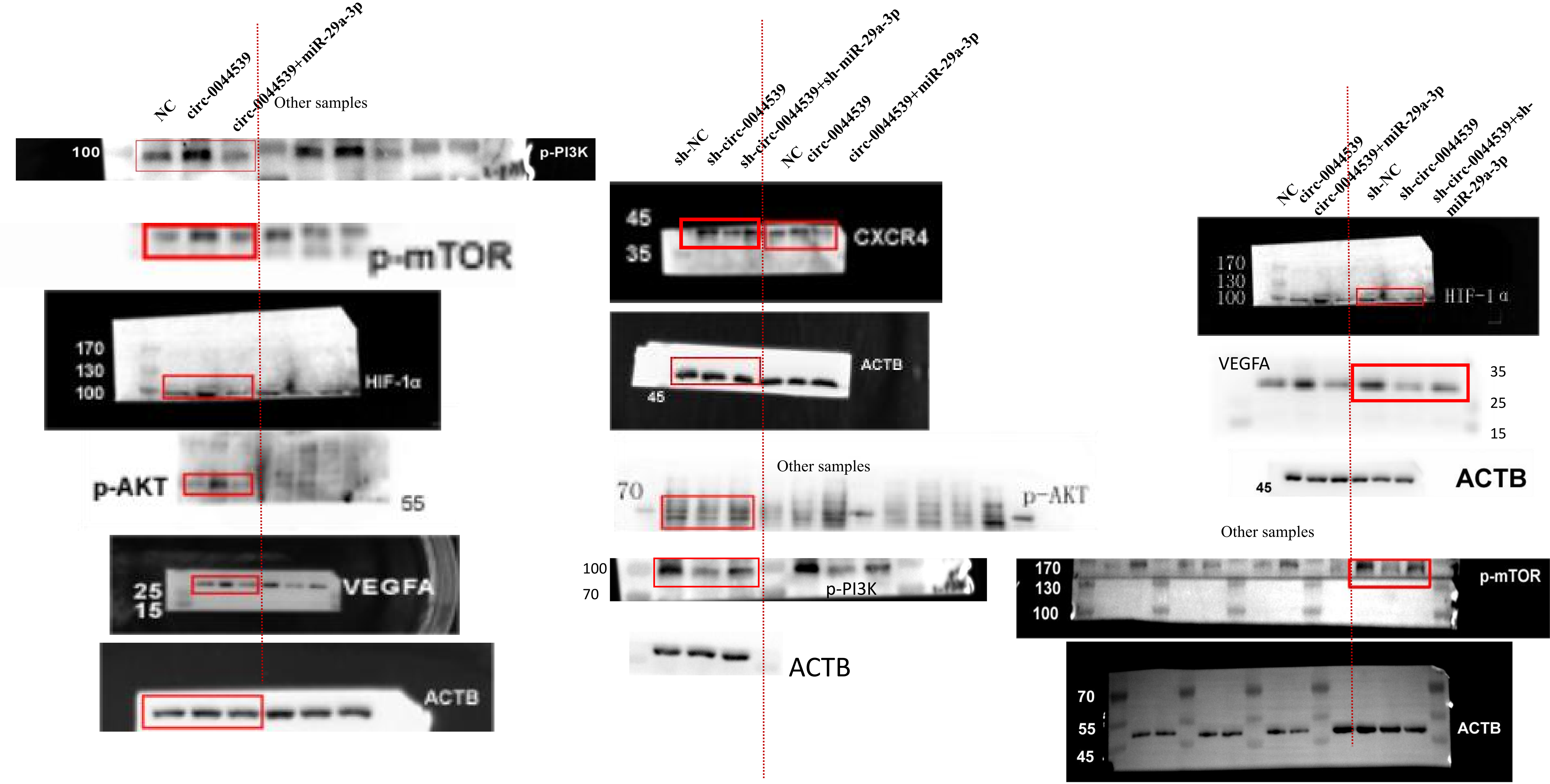

Unprocessed gels of Figure 4E

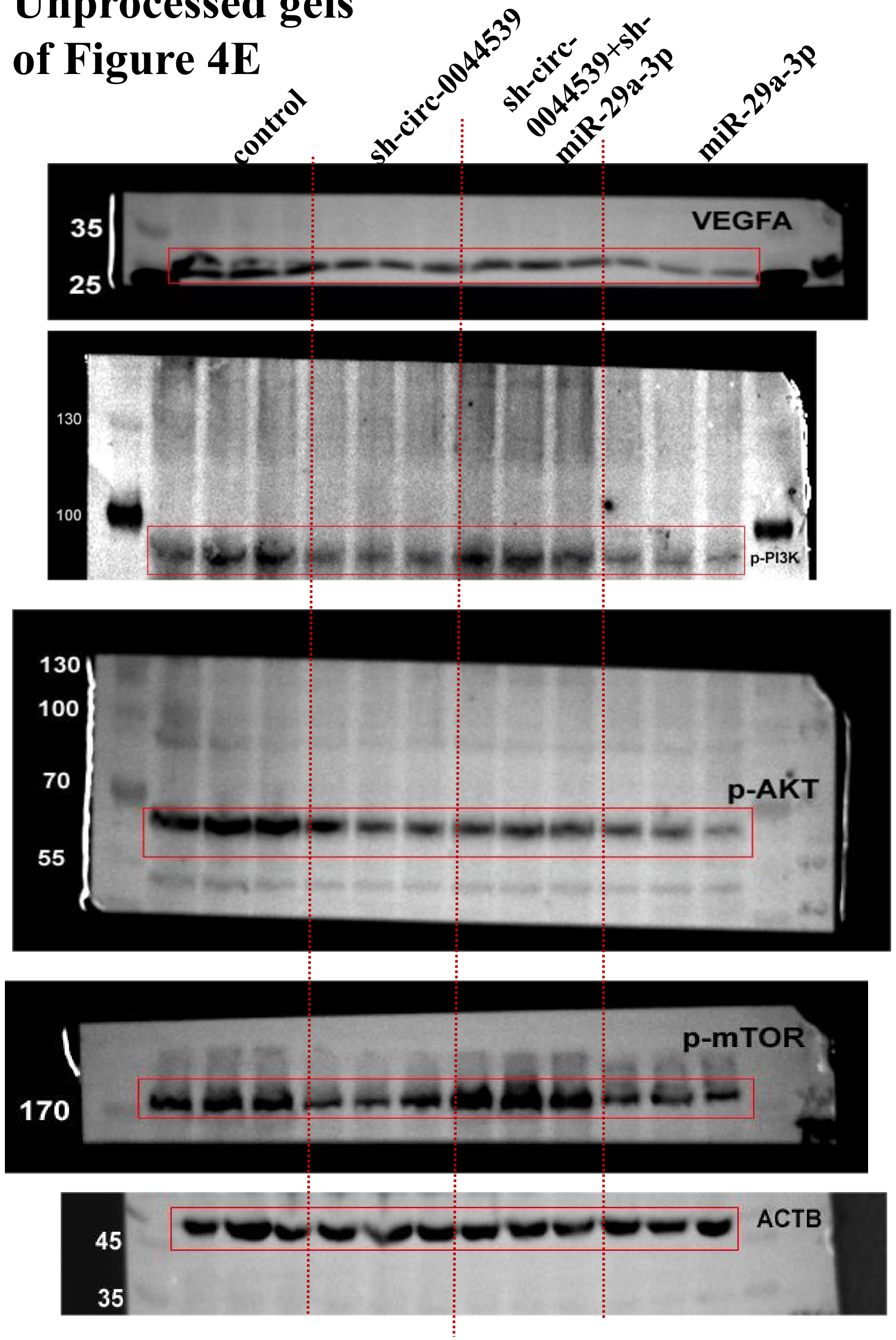

Unprocessed gels of Figure 5A

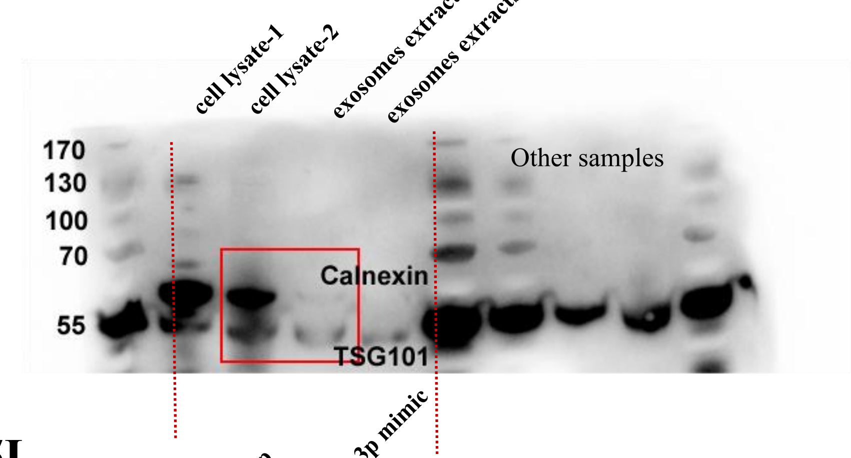

Figure 5I

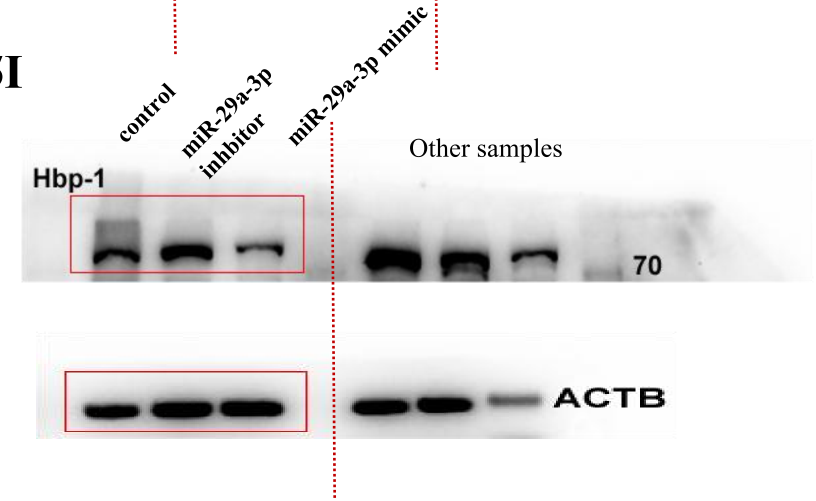

Unprocessed gels of Figure 7E

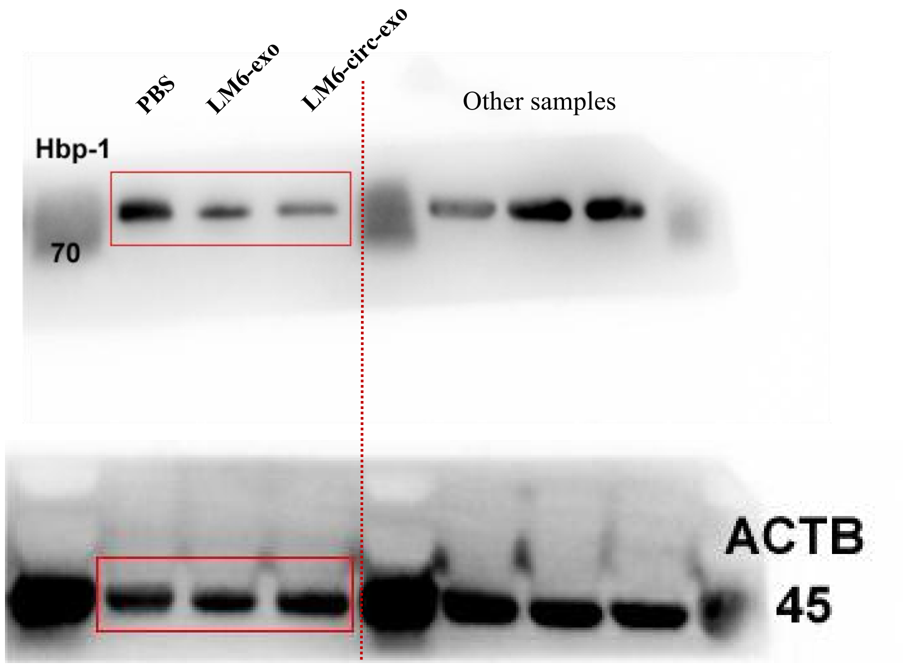

Unprocessed gels of Figure S4B: MHCC97L

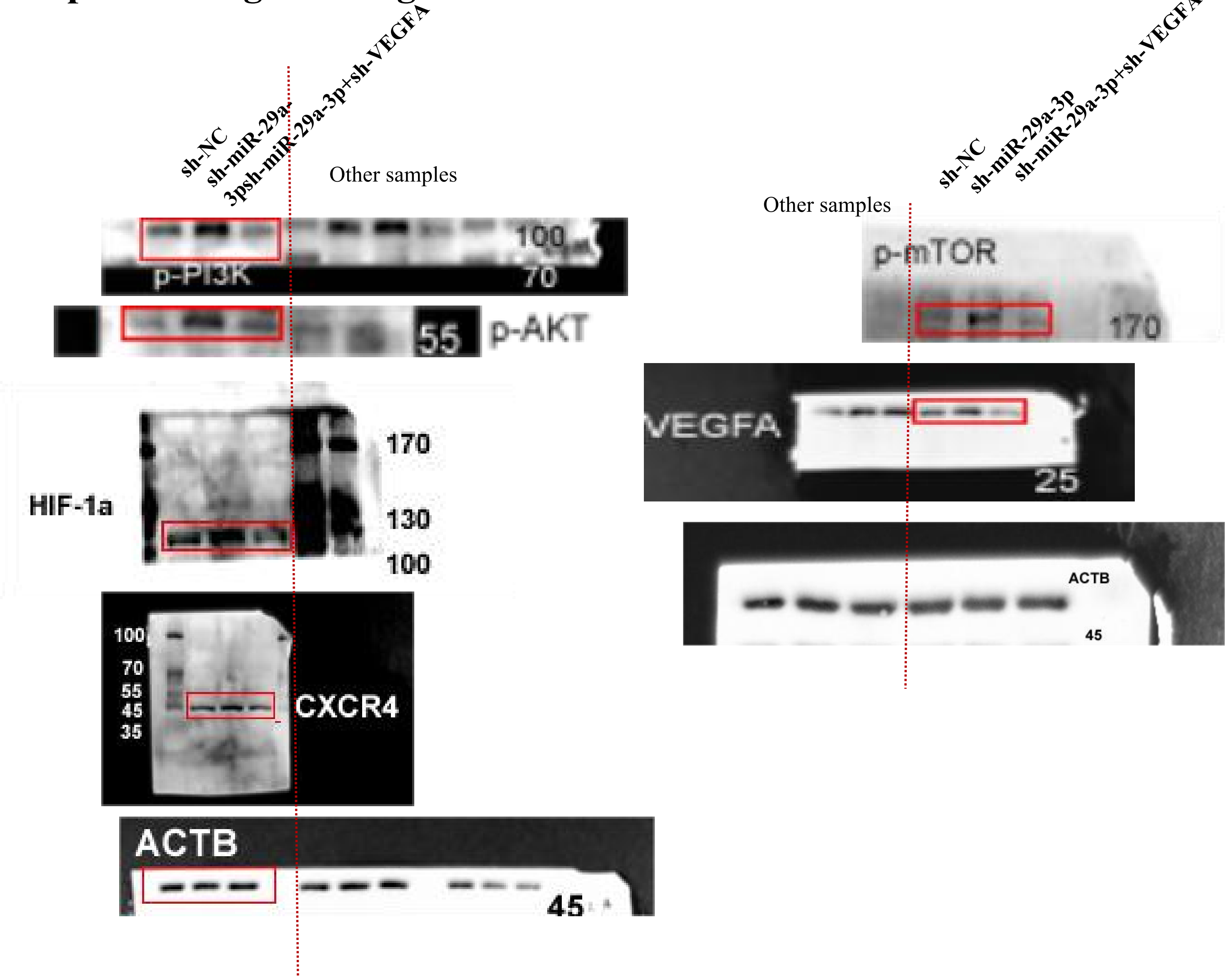

HCCLM6

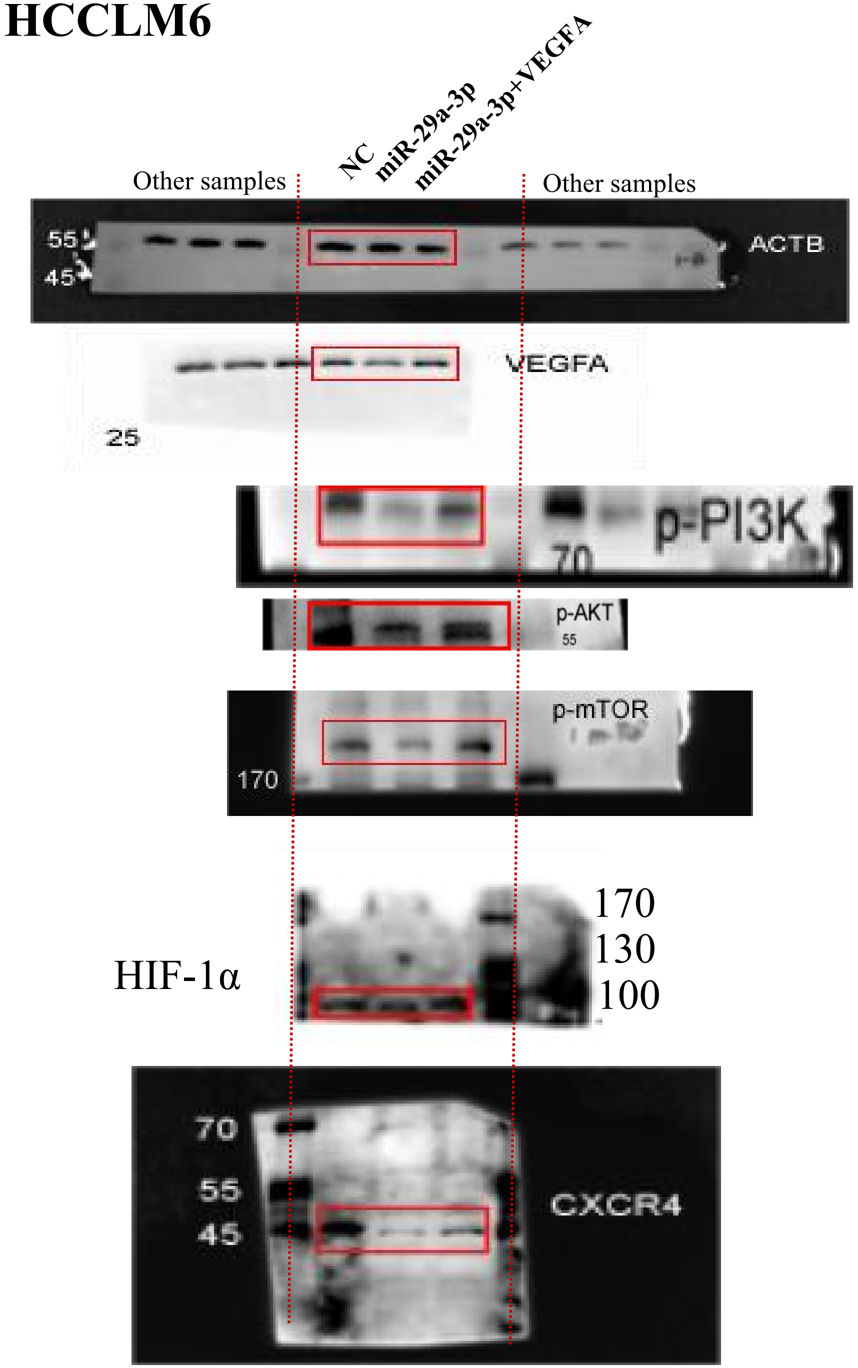

Supplement: Supplementary file 1 — Original Data File [file 41419_2024_7004_MOESM1_ESM.pdf]
