# Supplementary material for: Circ-0044539 promotes lymph node metastasis of hepatocellular carcinoma through exosomal-miR-29a-3p
Source: Cell Death Dis. 2024 Aug 27;15(8):630. doi: 10.1038/s41419-024-07004-x (PMC11349895; doi:10.1038/s41419-024-07004-x)
Supplement: Supplementary file 2 — Supplementary Materials [file 41419_2024_7004_MOESM2_ESM.pdf]

Supplementary Materials.

Cell culture

The human HCC cell lines HCCLM3, MHCC97H, HCCLM6 and MHCC97L, and the L02 and HEK293T cell lines, were generously provided by the Liver Cancer Institute, Zhongshan Hospital, Fudan University. HCCLM6 and MHCC97 were authenticated by STR. These cell lines were maintained in DMEM (HyClone, USA) supplemented with 10% fetal bovine serum (FBS) (Gibco, USA) and 1% penicillin-streptomycin-gentamicin (Beyotime, China). T cells were cultured in RPMI 1640 (HyClone, USA) with 10% FBS, 1% penicillin-streptomycin-gentamicin, and anti-CD3/CD28 beads (Thermo Fisher Scientific, USA). PMN-MDSCs were cultured in RPMI 1640 with 10% FBS, 1% penicillin-streptomycin-gentamicin and 5 μg/ml G-CSF (ACRO Biosystems, USA). All culture conditions were maintained at 37℃ in an incubator (Thermo Fisher Scientific, USA) with 5% CO_2. For hypoxia culture for exosome extraction, cells were exposed to in vitro hypoxic conditions using a hypoxia chamber with 1% O_2, 5% CO_2, and 94% N_2 at 37℃.

Lentivirus construction and cell transfection

The hU6-MCS-CBh-gcGFP-IRES-puromycin-shRNA-circ-0044539 lentiviral vectors, Ubi-MCS-SV40-EGFP-IRES-puromycin-circ-0044539 lentiviral vectors, hU6-MCS-Ubiquitin-EGFP-IRES-puromycin-hsa-miR-29a-3p inhibitor lentiviral vectors, Ubi-MCS-SV40-EGFP-IRES-puromycin-hsa-miR-29a-3p mimic lentiviral vectors, hU6-MCS-CBh-gcGFP-IRES-puromycin-VEGFA-RNAi lentiviral vectors Ubi-MCS-3FLAG-SV40-EGFP-IRES-puromycin-VEGFA lentiviral vectors, and hU6-MCS-CBh-gcGFP-IRES-puromycin-HIF-1α-RNAi lentiviral vectors were purchased from Shanghai Genechem Ltd. Circ-0044539-specific shRNAs were synthesized, specifically targeting the junction sequence. The short hairpin RNA (shRNA) target sequence of circ-0044539, hsa-miR-29a-3p, VEGFA and HIF-1α were shown in Supplementary Table S1. Treated cells were cultured with 5 μg/mL puromycin for three generations. Stably transfected clones were confirmed by qRT-PCR.

HCCLM6 cells were transfected with lentiviral vectors containing shRNA-circ-0044539-1, shRNA-circ-0044539-2, or shRNA-circ-0044539-3 to reduce circ-0044539 level. Among the shRNA-circ-0044539 constructs, shRNA-circ-0044539-1 exhibited the highest knockdown efficiency and was therefore selected for subsequent investigations (Fig.S2E).

Quantitative real-time PCR (qRT-PCR)

Total RNA was extracted using TRIzol reagent (Invitrogen, USA). RNA concentration and purity were measured by NonoDrop (Thermo, USA). Reverse transcriptase cDNA synthesis kit (Takara, Japan) was used, and qRT-PCR was performed with SYBR qPCR Master Mix (Yeasen, China) using Q6 Real-Time PCR System (Applied Biosystems, USA) according to the manufacturer's instructions. The results were normalized to β-actin (ACTB), or U6, and comparative quantification was assessed using the $2^{-\Delta\Delta Ct}$ method. All reactions were performed in triplicates. All primers used in this article are listed in Table S1.

Western blotting

Protein were extracted using RIPA buffer (NCM Biotech, China) with protease inhibitors and phosphatase inhibitor (NCM Biotech, China), added to 5× loading buffer (Beyotime, China). The

proteins were separated on a 10% sodium dodecyl sulfate polyacrylamide gel electrophoresis (SDS-PAGE), and transferred onto polyvinylidene difluoride (PVDF) membranes. 5% BSA was used for blocking the membrane for 1h at room temperature (RT), then the membrane was incubated with primary antibodies at 4°C overnight and followed by incubation with corresponding secondary antibodies for 1h at RT, detected by enhanced chemiluminescence assays. The primary antibodies used were shown in Table S2.

CCK-8 and colony formation assays

The proliferation of HCC cells was assessed using CCK-8 kit (Beyotime, China) assays. The cells were cultured in a 96-well plate at a density of 1000 cells in 100 µl of medium containing 10% FBS per well. The manufacturer's instructions were followed to generate a working solution by adding 10 µl of CCK-8 reagent to 90 µl of DMEM. Subsequently, 100 µl of the working solution was added to each well and incubated for 2 h. The absorbance of each well was measured at 450 nm, and the data obtained is presented as a line graph. The assay was conducted at 0 h, 24 h, 48 h, 72 h, and 96 h. All experiments were performed in triplicate.

For the colony formation assay, cells were digested into a single cell suspension, seeded with 1000 cells in each culture well (6-well plate) and added to an appropriate amount of complete medium, and the medium was changed every three days until the cells formed monoclonal colonies. Cells were then washed with PBS, fixed with 4% paraformaldehyde, and stained with 0.4% crystal violet for 15 min. The number of colonies containing >10 cells was counted manually. All experiments were performed in triplicate.

Transwell migration and invasion assay

The migration and invasion ability of HCC cells was assessed by transwell assay using a 24-well insert, with an 8 µm pore size with or without Matrigel (Corning, USA) according to the manufacturer's directions. In brief, 500 µL DMEM supplemented with 10% FBS was added to the lower chamber, while 1×10^5 cells in 200 µl FBS-free DMEM were seeded into the upper chamber. After culturing at 37 °C for 48 hours, a cotton swab was used to remove the cells from the upper surface of the chambers, and cells that penetrated to the underside of the membrane were fixed in 4% paraformaldehyde, stained with crystal violet (Beyotime, China), and counted in random fields under a microscope. All experiments were performed in triplicate.

Dual-luciferase reporter assay

A luciferase reporter assay was conducted using a dual-luciferase reporter gene detection kit (Promega, USA). The sequences of circ-0044539-3'UTR, VEGFA-3'UTR, Hbp1-3'UTR, and their miR-29a-3p binding site mutant versions were synthesized and added to the luciferase reporter vector. HCCLM6 or HEK293T cells were seeded into 24-well plates and cotransfected with circ-0044539-3'UTR, VEGFA-3'UTR, or HBP1-3'UTR, and miR-29a-3p mimics or miR-NC. After 48 h of incubation, Firefly and Renilla luciferase activities were examined. All experiments were performed in triplicate.

RNase R treatment and actinomycin D assay

Total RNA of HCCLM6 cells was incubated for 30 min at 37 ℃ with or without 5 U/μg RNase R (Epicenter Technologies, USA), and subsequently the abundance of linear RNA and circular RNA was determined by qRT-PCR.

For the actinomycin D assay, HCC cells were exposed to 5 μg/ml actinomycin D (Merck, Germany) at a series of time intervals (0, 4, 8, 12, and 24 h). Cells were harvested, and total RNA was extracted. The expression of circ-0044539 and COL1A1 mRNA was detected using qRT-PCR. All experiments were performed in triplicate.

RNA fluorescent in situ hybridization (FISH)

To detect circ-0044539 and miR-29a-3p, FISH was carried out with specific antisense probes of circ-0044539 and miR-29a-3p labeled with digoxin, purchased from Servicebio Company.

Cells were cultured on glass cover slips in 24-well plates for 24 h, and then fixed in 4% paraformaldehyde fix solution diluted with diethyl pyrocarbonate for 30 min at RT. Triton-X-100 in PBS was used to incubate the fixed cells for 5 min at RT. Then, the cells were incubated with pre-hybridization buffer for 30 min at 37 ℃ prior to the addition of probes in hybridization buffer at 37 ℃ overnight. Subsequently, wash cells with 4×saline-sodium citrate (SSC), 2×SSC and 1×SSC for 5 min and 3 times at 42 ℃. DAPI (Beyotime, China) was added to each slide and incubated for 5 min to stain the nuclei at RT. Then, we used microscopy to observe the subcellular location of circ-0044539 and miR-29a-3p.

Arginase activity assay, nitric oxide (NO) measurement, and ROS detection

Arginase activity was determined using an arginase assay kit according to the manufacturer's instructions (BioAssay Systems, CA). Briefly, lyse cell pellets for 10 min in 100 μl of 10 mM Tris-HCl (pH 7.4) and collect supernatant for arginase assay after centrifuging lysates at 14,000 g at 4°C for 10 min. Then, Substrate Buffer combined with Arginine Buffer were added, and mixture was incubated 1 h at RT and read optical density at 430 nm.

NO was measured using a Griess reagent kit (Beyotime, China). According to the manufacturer's instructions, culture supernatant (50 μl) was mixed with Griess reagent, and incubated for 30 min at RT. The absorbance of each sample was measured at 540 nm in a microplate reader (Thermo Fisher, USA). The NO concentration can be acquired according to their calibration curves.

An ROS fluorescent labeling assay (Beyotime, China) was used to assess intracellular ROS levels. PMN-MDSCs were co-cultured with HCC exosomes for 1 day, and incubated with a fluorescent DCFH-DA probe at 37°C for 30 min in the dark. A microplate reader was utilized to detect DCFH-DA fluorescence at 525 nm. All experiments were performed in triplicate.

Detection of lactate dehydrogenase (LDH) release

The LDH Release Assay Kit was used to determine the level of LDH released into the cell culture supernatants according to the manufacturer's instructions (Beyotime, China). A microplate reader was used to measure the absorbance OD value at 490 nm. All experiments were performed in triplicate.

HCC subcutaneous xenografted nude mice model and liver orthotopic HCC in nude mice

Male BALB/c nude mice (4 weeks of age, 18–20 g of weight) were used for xenograft models and maintained under SPF conditions in accordance with a protocol approved by the Animal Ethics Committee of Tongji University. Nude mice were purchased from GemPharmatech (Jiangsu, China), and housed under standard conditions in the animal care facility at the Experimental Animal Center of Shanghai East Hospital, Tongji University. The mice were randomly divided into four groups. HCCLM6-luciferase cells with alternations of circ-0044539 and miR-29a-3p (1×10^6 cells in 0.1 ml of PBS) were subcutaneously injected into nude mice (n = 5/group). Tumor growth was monitored twice a week. At week 5, the nude mice were sacrificed. The tumor weight and volume were measured. Volume was calculated as follows: tumor volume (mm3)=0.5×length×width^2. In addition, protein samples from the tumor were extracted for Western blot analysis of VEGFA-PI3K-AKT-mTOR protein expression.

The liver orthotopic HCC implant tumor model in nude mice was performed as previously described[1]. The subcutaneous tumor tissues were removed and implanted into the liver of nude mice and examined by BLI assay. After 6 weeks of liver implantation, the mice were sacrificed, and tumors were separated from liver tissue. The live tumors, LNs of mice were fixed and serially sectioned. Then, LNs were stained with HE to identify metastatic lesions. Tumors were stained through IHC to detect VEGFA, HIF-1α and CXCR4 expression in these groups. All surgical procedures and care administered to the animals were in accordance with the institutional ethics guidelines.

Construction of HCC cell lines stably secreting near-infrared fluorescence protein 682 (iRFP682)-labeled exosomes

By cloning the iRFP682 gene and exosomal marker protein CD63 gene into a plasmid, coating with lentivirus, and transfecting HCC cell lines, we constructed HCC cell lines that stably expressed the iRFP682 and CD63 fusion protein. On this basis, the circ-0044539 overexpression lentivirus was transfected into HCCLM6 cells, which could stably release exosomes labeled with iRFP682. Through exosome isolation and purification, iRFP682-labeled exosomes were used in further studies of the distribution and signal transduction of exosomes in vivo.

Human immune system reconstitution NSG mice (Hu-NSG)

Hu-NSG were generated using NOD-Prkdc^{scid}Il2rg^{em1}Hc$^{em3(c.645InsTA)Smoc}$ (NSG) mice, irradiated (150 cGy) and injected with human CD34+ cord blood stem cells. Mice were bled after transplantation, and then flow cytometry was used to determine the extent of reconstitution with human leukocytes[2]. At least 25% of CD45+ leukocytes were human CD45+. Hu-NSG were raised under specific pathogen-free conditions for 12 weeks. The animal experiment received approval from the Animal Ethics Committee of Tongji University. The Hu-NSG mice (16 weeks of age, male) were randomly assigned into three groups, and each group was treated with either PBS, HCCLM6-exosomes, or HCCLM6-circ-0044539-exosomes.

Consturcting Hu-NSG model of pre-metastasis LNs niche

The Hu-NSG model of pre-metastatic LN environment was established by injection of HCC exosomes into the right footpad of Hu-NSG. HCC exosomes (5 μg/40 μl) were injected into each Hu-NSG, three times a week for a total of 4 weeks. On the days after exosome inoculation, the iRFP682 signal of exosomes was examined to show the process of exosome migration. In the first

week, three mice from each group were sacrificed. The frequencies of PMN-MDSCs in spleen, LN and blood, and Hbp1 and Arg1 expression in LN cells were evaluated.

In the 4 weeks of HCC exosomes inoculation, 5×10^6 HCCLM6-Luc cells were injected into the right footpad of the Hu-NSG. Metastasis to the LN was assessed using in vivo BLI (IVIS Spectrum, USA) at weeks 2, 4, and 6 after injection of HCCLM6-Luc cells.

Footpad injection

Reportedly, HCCLM6 cells had a tumor formation rate of 100% and LNM rate of 75% in a nude mice footpad inoculation model at week 5[3]. A volume of 40 µl DMEM containing 5 µg exosomes or 5×10^6 HCCLM6-Luc cells was slowly injected into the right footpad of the Hu-NSG using a 100 µl syringe and 30 G needle to prevent leakage to the intermuscular spatium. In addition, the methylene blue dye indicator showed movement from the footpad to the popliteal LN in this injection method.

LN separation, LN cell isolation and LNM evaluation

In Hu-NSG, popliteal LNs were harvested in PBS on ice, and subjected to mincing and digestion with Collagenase D (Roche, Swiss) in RPMI-1640 for 20 minutes at 37℃ prior to mechanical dissociation on 220 mesh cell strainers (68 µm)[4]. LN cells were washed with PBS supplemented with 1% FBS.

Hu-NSG were euthanized 6 weeks following HCCLM6-Luc injection. Popliteal, inguinal, sciatic, and axillary LNs were evaluated for their gross feature. The embedded specimens were cut into 4-mm-thick serial sections and stained with HE.

In the liver orthotopic HCC nude mice model, the ratio of LNM=the number of mice with LNM /total 5 mice in each group.

In Hu-NSG, the ratio of LNM=the number of LNM determined by HE/12 (four LNs of each mouse: popliteal, inguinal, internal iliac and axillary LNs. Three Hu-NSG in each group).

BLI assay and fluorescence imaging assay.

The BLI assay was performed using D-Luciferin (30 mg/mL, 770505, PerkinElmer). For in vivo imaging, mice were injected intraperitoneally (i.p.) with a fresh stock solution of D-Luciferin in DPBS (150 mg/kg body weight). The luminescence signal around the body of the mice was measured 10 min after mice were anesthetized with 1% pelltobarbitalum natricum (50 mg/kg), and at 10 min after D-Luciferin injection. In vivo BLI assays were serially performed 6 weeks after liver surgery in nude mice or 2, 4 and 6 weeks after footpad inoculation in Hu-NSG. Images of the animals were acquired using Living Image software (Xenogen, CA). For fluorescence imaging, image were obtained using a 745 nm excitation and 800 nm emission filter set. Imaging signals were collected in an IVIS (PerkinElmer, USA).

Animal studies of exosomes are constrained by limitations in the techniques available for noninvasive monitoring of the progression of exosome movement. Because the size of mouse LNs is 1 to 2 mm, changes in the internal structure of murine LNs from the onset of metastasis cannot be detected easily. Our results showed that exosomes fused with iRFP682 might be an optimal method to evaluate exosome migration. However, intact skin blocks the fluorescence signal, and spontaneous fluorescence of hollow organs causes a relatively high background signal, which should be overcome in further research to show visceral LNs.

Reference

1. Gao YS, Chen XP, Li KY, Wu ZD. Nude mice model of human hepatocellular carcinoma via orthotopic implantation of histologically intact tissue. World J Gastroenterol. 2004;10(21):3107-11.
2. Najima Y, Tomizawa-Murasawa M, Saito Y, Watanabe T, Ono R, Ochi T, et al. Induction of WT1-specific human CD8+ T cells from human HSCs in HLA class I Tg NOD/SCID/IL2rgKO mice. Blood. 2016;127(6):722-34.
3. Li Y, Tian B, Yang J, Zhao L, Wu X, Ye SL, et al. Stepwise metastatic human hepatocellular carcinoma cell model system with multiple metastatic potentials established through consecutive in vivo selection and studies on metastatic characteristics. J Cancer Res Clin Oncol. 2004;130(8):460-8.
4. Lee H, Lee YS, Harenda Q, Pietrzak S, Oktay HZ, Schreiber S, et al. Beta Cell Dedifferentiation Induced by IRE1alpha Deletion Prevents Type 1 Diabetes. Cell Metab. 2020;31(4):822-36 e5.

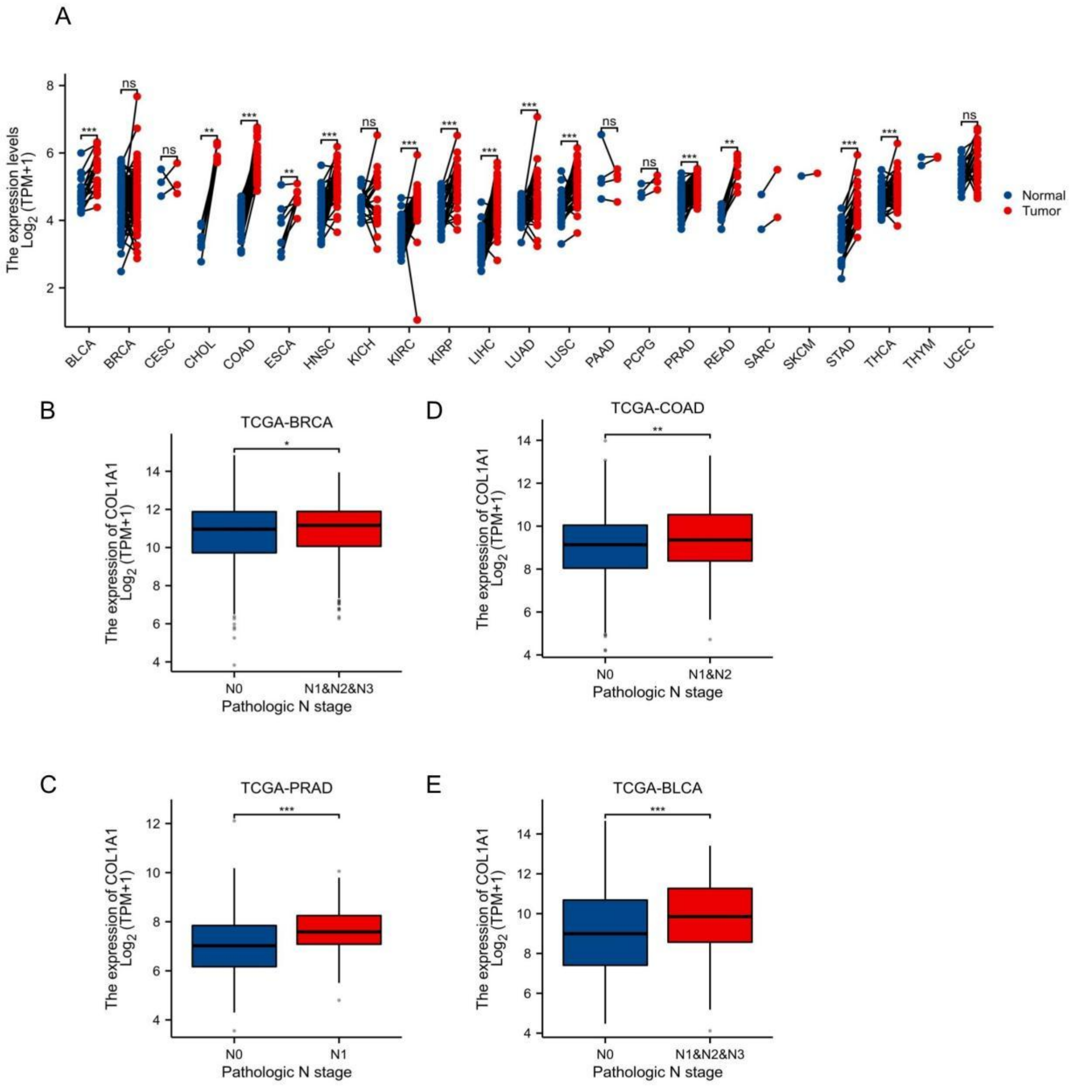

Figure S1. COL1A1 mRNA expression across cancers in the TCGA database.

A. The expression of COL1A1 mRNA in paired tumor and adjacent normal tissues in the indicated tumor types.

B-E. COL1A1 mRNA expression in different N stages in BRCA (B), PRAD (C), COAD (D) and BLCA (E).

*p < 0.05, ***p < 0.001, ****p < 0.0001 (Student's t-test).

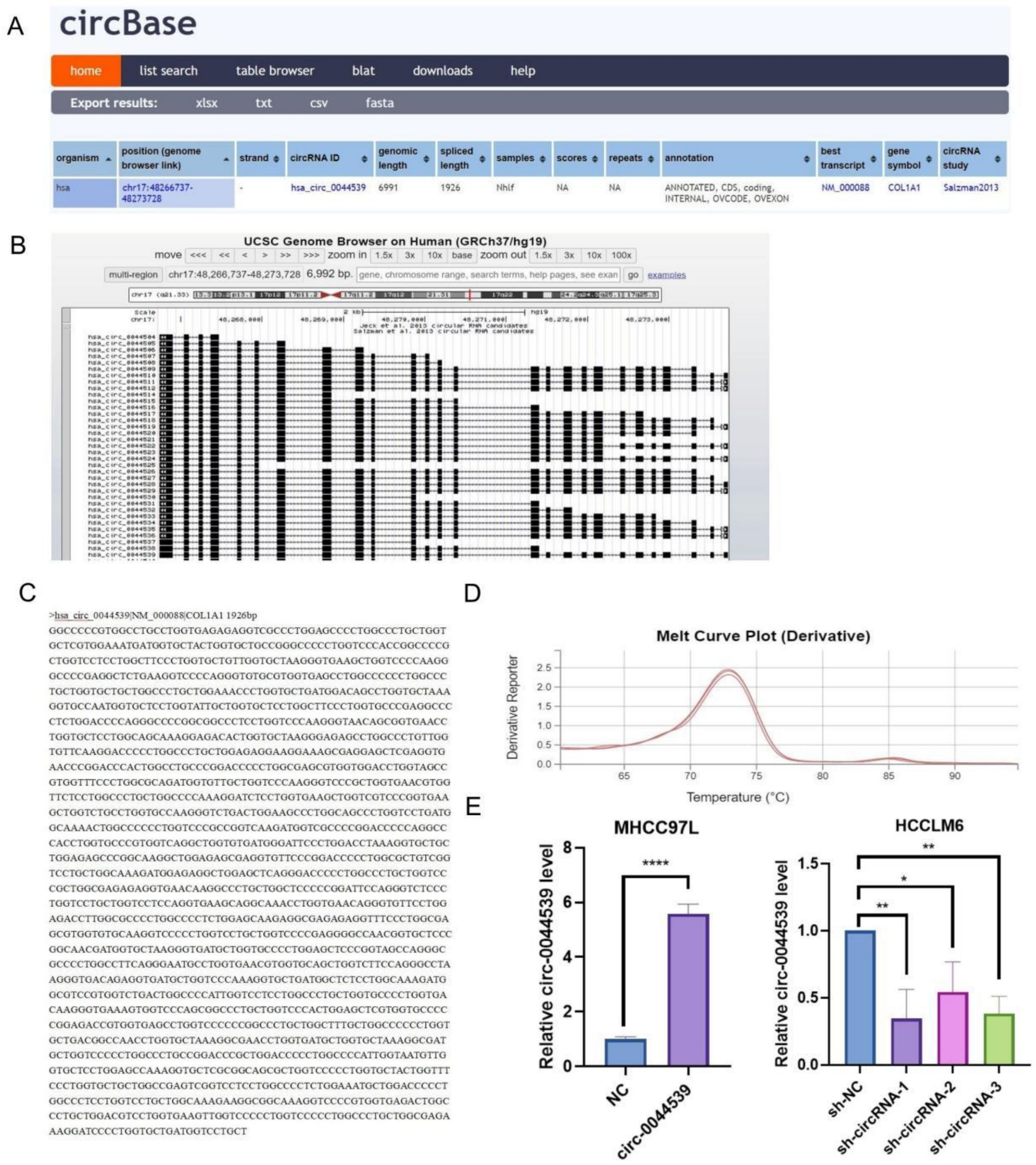

Figure S2. Chromosomal localization and biological information of circ-00044539.

A. Circ-0044539 in circBase.

B. Circ-0044539 in UCSC.

C. Sequence of the human circ-0044539 from UCSC.

D. Demonstration of primers of circ-0044359 with normal melt curves of single peaks.

E. qRT-PCR showed the transfection efficiency of circ-0044539 in MHCC97L and HCCLM6. Data presented as means ± SD of three independent experiments. *$p < 0.05$, ***$p < 0.001$, ****$p < 0.0001$ (Student's t-test).

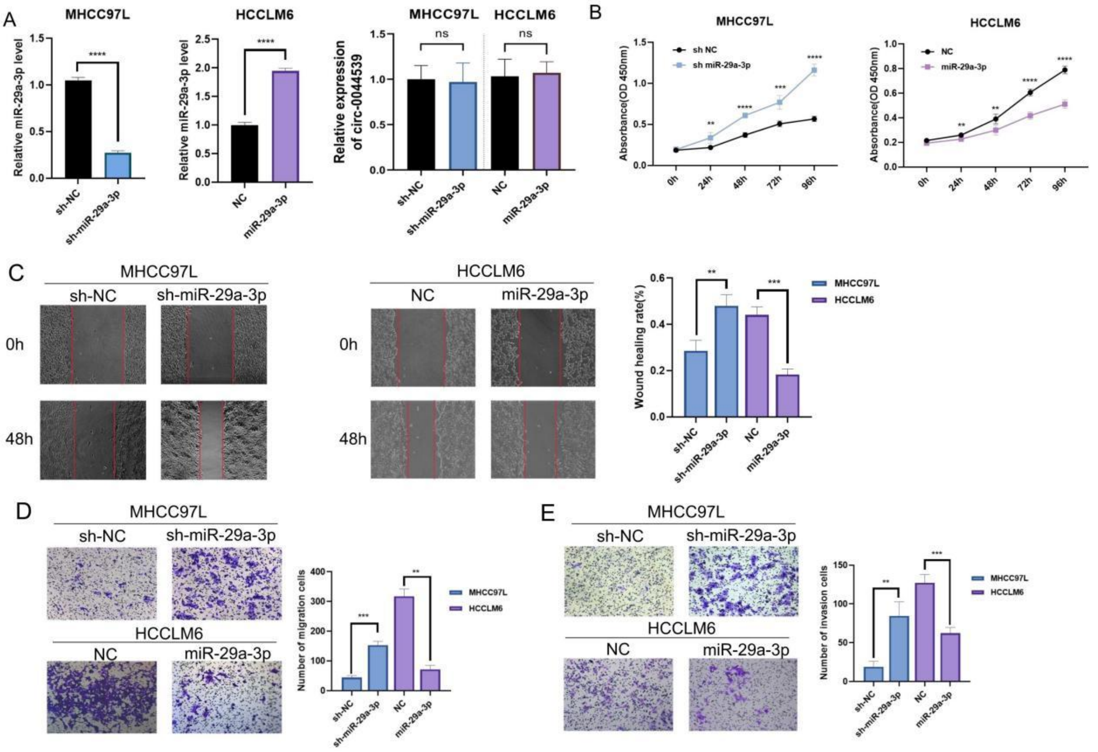

Figure S3. Impact of miR-29a-3p on HCC cells.

A. qRT-PCR showed the transfection efficiency of miR-29a-3p and the corresponding circ-0044539 expression in MHCC97L and HCCLM6.

B. The CCK-8 assay showed the influence of miR-29a-3p on the proliferation ability of MHCC97L and HCCLM6 cells.

C. Wound healing assay showed the influence of miR-29a-3p on the migration capacity of MHCC97L and HCCLM6 cells.

D-E. Transwell assays showed the influence of miR-29a-3p on the migration and invasion capacity of MHCC97L and HCCLM6 cells.

Data presented as means ± SD of three independent experiments. *$p < 0.05$, ***$p < 0.001$, ****$p < 0.0001$ (Student’s t-test).

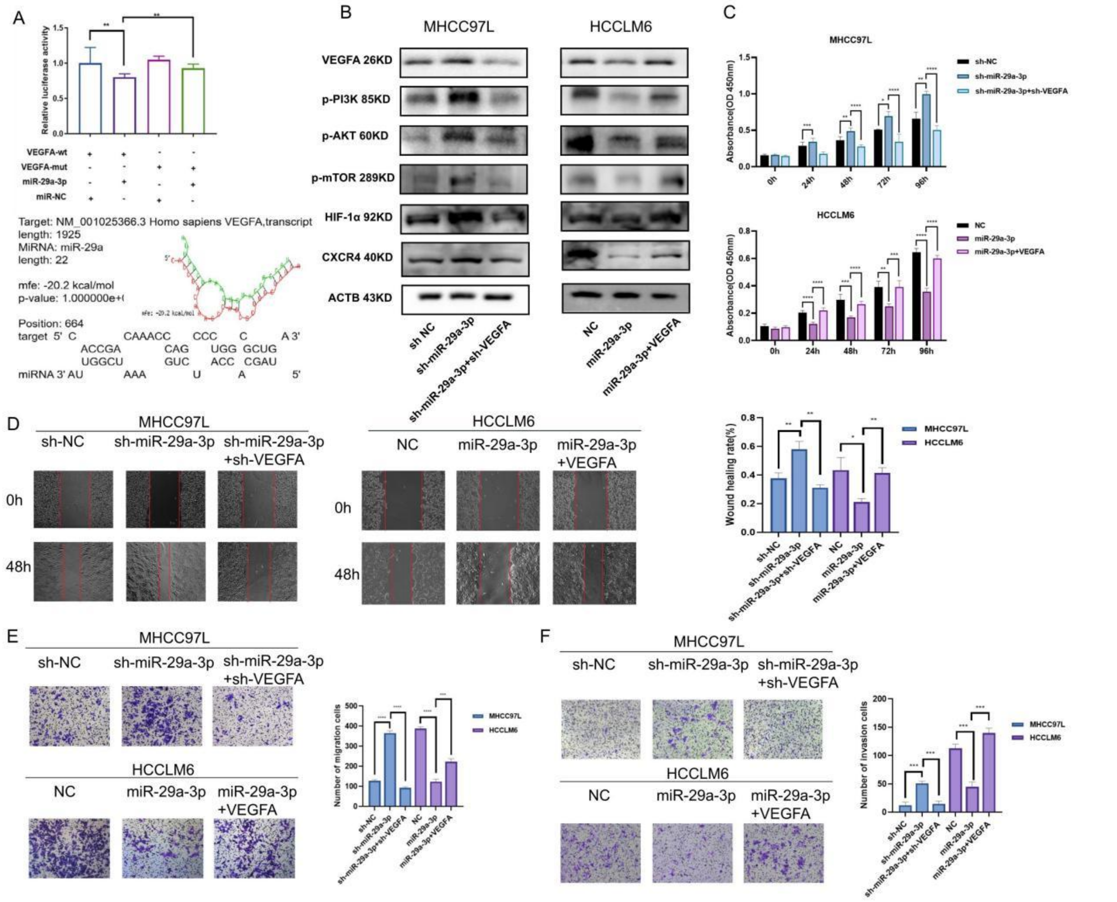

Figure S4. miR-29a-3p functions as a tumor suppressor through VEGFA in HCC cells.

A. Dual-luciferase reporter gene assay showed a direct interaction between miR-29a-3p and the VEGFA 3'UTR in HCCLM6.

B. Western blot analysis showed that miR-29a-3p suppressed downstream molecules that could be opposed by VEGFA.

C. CCK-8 analysis showed that VEGFA reversed the effect of miR-29a-3p on the proliferation in HCC cells.

D-F. Wound healing assay and transwell assays showed that VEGFA reversed the effect of miR-29a-3p on the migration and invasion in HCC cells.

Data presented as means ± SD of three independent experiments. $*p < 0.05$, $***p < 0.001$, $****p < 0.0001$ (Student's t-test).

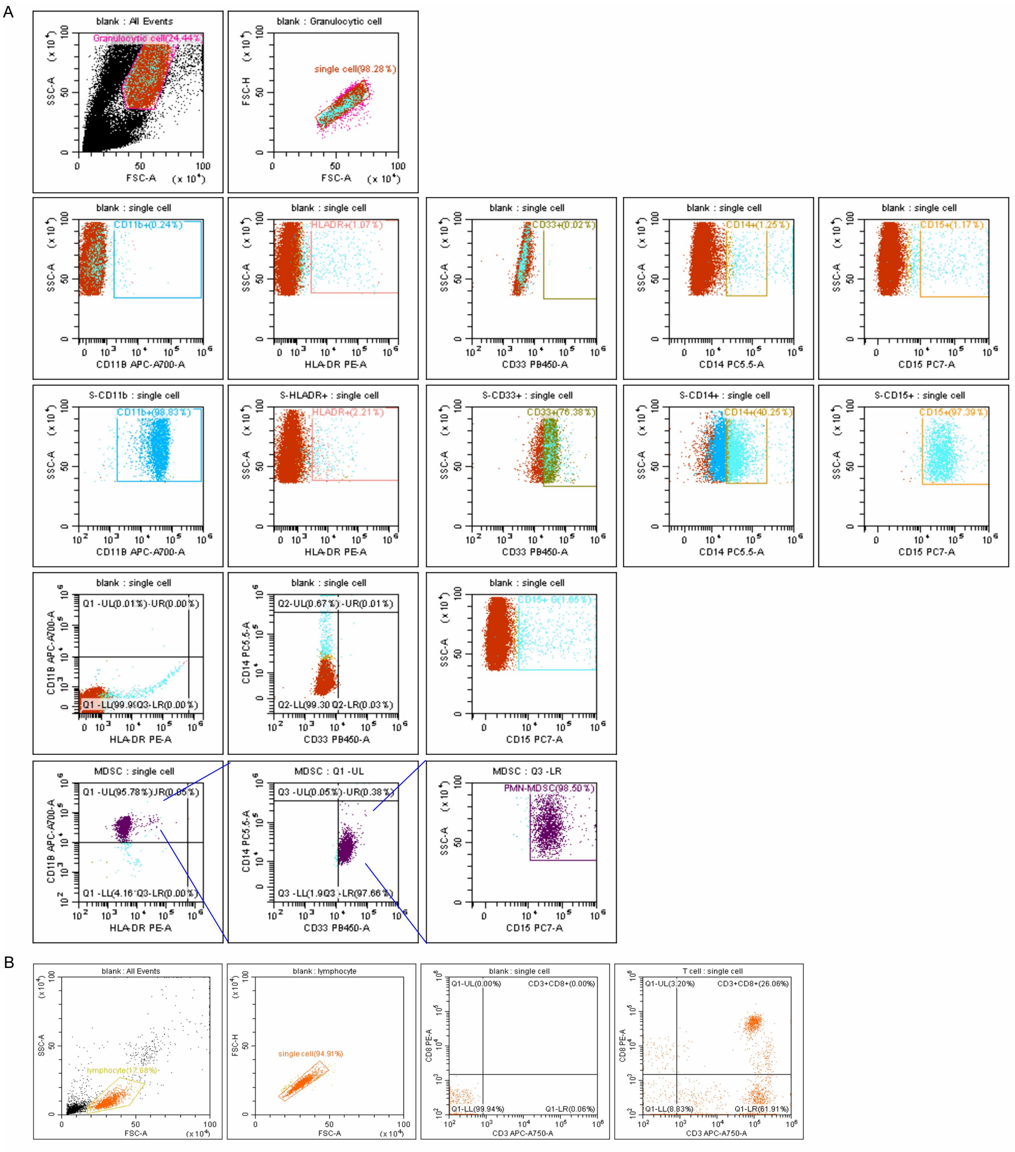

Figure S5. PMN-MDSCs and T cells extracted from human peripheral blood.

A. The gating strategy for the determination of circulating PMN-MDSCs (CD11b+HLA-DR−CD33+CD14-CD15+) is depicted.

B. Representative dot plots of CD3+CD8+ T cells in blood from healthy donors.

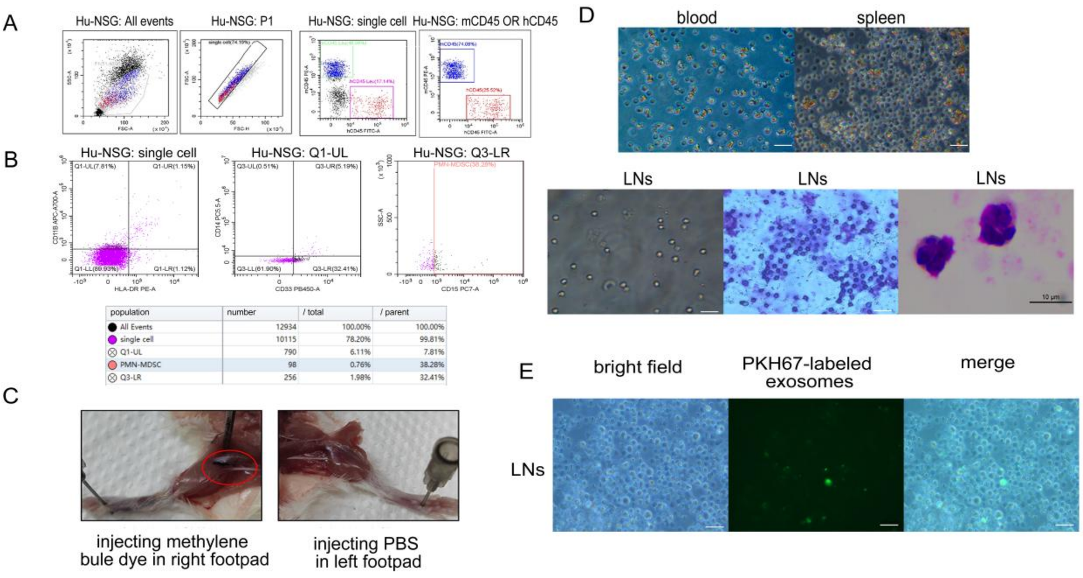

Figure S6. Construction of the Hu-NSG footpad inoculation model.

A. The percentage of human CD45+ cells in total CD45+ cells in the peripheral blood of Hu-NSG mice was measured.

B. The percentage of PMN-MDSCs in Hu-NSG peripheral blood was measured.

C. Injecting methylene blue dye into the Hu-NSG footpad showed the lymphatic drainage direction.

D. Extracting blood, spleen, and LNs from Hu-NSG. Giemsa staining showed the morphology of polymorphonuclear cells in LNs.

E. Injecting PKH67-labeled exosomes into the footpad led to enrichment of exosomes in the LN cells.

Table S1. Primer sequences in this study

Gene	Sequence
**Primers for qRT-PCR**	
circ-0044539	forward 5'-ACGTCCTGGTGAAGTTGGTC-3'
	reverse 5'- CATTTCCACGAGCACCAGC-3'
miR-29a-3p	forward 5'-CCGTAGCACCATCTGAAATCGGTTA-3'
VEGFA	forward 5'-AGGGCAGAATCATCACGAAGT-3'
	reverse 5'-AGGGTCTCGATTGGATGGCA-3'
Hbp-1	forward 5'-TCATCACCATTGGAAGGAGGA-3'
	reverse 5'-TTGCACCATCCCAAATCATCA-3'
U6	forward 5'-GGAACGATACAGAGAAGATTAGC-3'
	reverse 5'-TGGAACGCTTCACGAATTTGCG-3'

HIF-1α	forward 5'-GAACGTCGAAAAGAAAAGTCTCG-3'
	reverse 5'-CCTTATCAAGATGCGAACTCACA -3'
ACTB	forward 5'-CATGTACGTTGCTATCCAGGC-3'
	reverse 5'-CTCCTTAATGTCACGCACGAT-3'
**Target sequence of lentivirus or plasmid construction**	
shRNA-circ-0044539-1	5'-ATGGTCCTGCTGGCCCCCGTG -3'
shRNA-circ-0044539-2	5'-TCCTGCTGGCCCCCGTGGCCT-3'
shRNA-circ-0044539-3	5'-GATGGTCCTGCTGGCCCCCGT-3'
shRNA-miR-29a-3p	5'-TAACCGATTTCAGATGGTGCTA-3'
VEGFA-RNAi	5'-GCCAGCACATAGGAGAGATGA-3'
shRNA-HIF-1α	5'-AATGTGAGTTCGCATCTTGAT-3'

Table S2. Primary antibodies used in western blot, immunohistochemistry and flowcytometer in this study

Antibody	Test	Concentration	Company	Code
VEGFA	WB	1:1000	Cell Signaling Technology	50661
	IHC	1:200	Beyotime	AF1309
Phospho-PI3K	WB	1:1000	Cell Signaling Technology	17366
Phospho-AKT	WB	1:2000	Cell Signaling Technology	4060
Phospho-mTOR	WB	1:1000	Cell Signaling Technology	5536
HIF-1α	WB	1:1000	Beyotime	AF7087
	IHC	1:400	Cell Signaling Technolog	48085
CXCR4	WB	1:1000	Beyotime	AF6621
	IHC	1:800	Cell Signaling Technolog	97680
TSG101	WB	1:1000	Santa cruz	sc-7964
Calreticulin	WB	1:1000	Santa cruz	sc-373863
Hbp-1	WB	1:1000	Santa cruz	sc-515281
CD3	Flow cytometry	1:20(5/100 µl)	BD Pharmingen	557832
CD8	Flow cytometry	1:5 (20/100µl)	BD Pharmingen	555635
CD14	Flow cytometry	1:20(5/100 µl)	BD Pharmingen	566465
HLA-DR	Flow cytometry	1:5 (20/100µl)	BD Pharmingen	555812
CD33	Flow cytometry	1:20(5/100 µl)	BD Pharmingen	562854
CD11b	Flow	0.5 mg/ml	BD Pharmingen	557396

CD15	cytometry<br>Flow<br>cytometry	1:20(5/100 µl)	BD Pharmingen	560827

Table S3. Predictive analysis of miRNA response elements (MREs) of circRNAs. (The number after $ represents the number of microRNA binding sites)

circRNA ID	host gene	circRNA_MRE1	circRNA_MRE2	circRNA_MRE3
hsa_circ_0044534	COL1A1	hsa-miR-4640-5p$20	hsa-miR-4726-5p$20	hsa-miR-4733-3p$19
hsa_circ_0044539	COL1A1	hsa-miR-29b-3p$20	hsa-miR-29a-3p$20	hsa-miR-29c-3p$20
hsa_circ_0044542	COL1A1	hsa-miR-29b-3p$3	hsa-miR-29a-3p$3	hsa-miR-29c-3p$3
hsa_circ_0044547	COL1A1	hsa-miR-4733-3p$15	hsa-miR-4640-5p$15	hsa-miR-4726-5p$15
